# The Endocannabinoid–Microbiota–Neuroimmune Super-System: A Unifying Feedback Architecture for Systems Resilience, Collapse Trajectories, and Precision Feedback Medicine

**DOI:** 10.3390/ijms262210959

**Published:** 2025-11-12

**Authors:** Cătălin Aliuș, Alexandru Breazu, Cosmin Pantu, Corneliu Toader, Matei Șerban, Răzvan-Adrian Covache-Busuioc, Octavian Munteanu, Adrian Vasile Dumitru

**Affiliations:** 1 Faculty of General Medicine, “Carol Davila” University of Medicine and Pharmacy, 050474 Bucharest, Romania; catalin.alius@umfcd.ro (C.A.); dr.breazualexandru@gmail.com (A.B.); 2 Department of Anatomy, “Carol Davila” University of Medicine and Pharmacy, 050474 Bucharest, Romania; 3 Puls Med Association, 051885 Bucharest, Romania; 4 Department of Neurosurgery, “Carol Davila” University of Medicine and Pharmacy, 020021 Bucharest, Romania; 5 Department of Vascular Neurosurgery, National Institute of Neurology and Neurovascular Diseases, 077160 Bucharest, Romania; 6 Department of Pathology, Faculty of Medicine, “Carol Davila” University of Medicine and Pharmacy, 030167 Bucharest, Romania

**Keywords:** endocannabinoid system, gut microbiota, neuroimmune interface, systems resilience, precision feedback medicine, collapse trajectories, multi-omics biomarkers, digital twins

## Abstract

Modern biomedicine frequently contextualizes disease around isolated molecular or organ-specific mechanisms, but numerous chronic diseases, including Alzheimer’s disease, multiple sclerosis, depression, diabetes, and sepsis, share common trajectories of systemic destabilization. An increasing body of evidence indicates that health is not a property of single organs but the emergent property of interdependent feedback networks linking the microbiome, endocannabinoidome, neuroimmune system, and metabolic regulators. We propose the Endocannabinoid–Microbiota–Neuroimmune Super-System (EMN-S) as an evolutionarily conserved conceptual model that describes how these fields of influence reciprocally interact through feedback control. The microbial communities constituting the EMN-S encode environmental and dietary inputs, endocannabinoid signaling serves as an integrative regulator that synchronizes neural and immune activity, and neuroimmune circuits effectuate adaptive behaviors that alter microbiotal and lipid ecosystems. This review formalizes the EMN-S, contending that it is a unitary and cohesive model of physiological resilience, as well as offering a framework for precision feedback therapeutics. We describe how three mechanisms—encoder drift, integrator detuning, and executor overutilization—convert stabilizing negative feedback into runaway feedback cascades that underlie chronic, recurrent, and multisystemic disease. We then specify the EMN-S signature—integrated microbiome, lipidomic, and immune readouts—as an early indicator of resilience collapse and prospective preclinical state. Finally, we recapitulate the potential of AI-driven digital twins to illuminate feedback collapse, predict tipping points, and direct closed-loop intervention and treatments to restore dynamic equilibrium. By anchoring complexity in concrete and measurable feedback principles, the EMN-S shifts focus to investigate pathophysiology as opposed to reductionist lesion models of systemic derangements and embraces a systemic, empirically testable theory of stability.

## 1. Introduction: The Missing Neuro-Super-System

For much of the era of modern neuroscience, the brain has been studied in isolation, with function over-interpreted as innate to the dynamic interactions of neural circuits, synaptic connections, and co-activity of glia. This reductionistic model has indeed produced valuable insights into the mechanisms of neural operation, but it tells us nothing that can account for the rich variety and systemic interdependence that is observed in many neurological and psychiatric diseases or the ways in which they interact with overall human well-being [1]. For the past thirty years or so, a succession of converging discoveries has combined to undermine much of the strictly brain-centric view of its functional utility. Two major meta-regulatory domains have emerged as crucial regulators of brain–body communication: the gut microbiota and the endocannabinoid system (ECS) [2].

The gut microbiota was once viewed as a passive assemblage of commensal organisms but is now recognized as an active meta-organ, well-endowed functionally as an endocrine and immune entity with a wide-ranging capacity to produce a repertoire of bioactive metabolites with the capacity for direct neuromodulation. Short-chain fatty acids (SCFAs), tryptophan derivatives, bile acids, and microbially derived neuroactive compounds can now be seen to have effects on vagal signaling, blood–brain barrier integrity, neuro-inflammatory cascades, and even adult hippocampal neurogenesis [3,4]. Consequently, the concept of the gut–brain axis has revolutionized our perceptions of cognitive development, mood regulation, and the neuroplastic decline of the organism. Concomitant with such results, the ECS, originally proposed in the form of its paradigmatic ligands (i.e., anandamide and 2-arachidonoylglycerol) and receptors (i.e., CB1 and CB2) in the early 1990s, has developed into the broader eCBome, otherwise known as a network containing lipid mediators, receptors (GPR55, TRPV1, and PPARs), and enzymes involved in lipid metabolism [5]. This complicated system regulates various processes that maintain homeostasis and modulate key processes affecting energy control, immune tolerance, response to stress, and synaptic plasticity [6]. The eCBome is thus a paradigm shift: endocannabinoid signaling is not confined to neural circuitry but integrates neural, immune, and metabolic function. Unfortunately, microbiota research and eCBome research have historically developed in parallel, with limited cross-fertilization [7]. Microbiome studies have tended to concentrate on dysbiosis and its inflammatory or metabolic sequelae in illnesses such as Alzheimer’s disease, Parkinson’s disease, depression, and autism. The focus in ECS research, on the other hand, has been on receptor pharmacology and neuromodulation, with too little attention focused on microbial dynamics [8]. This, however, has begun to change, and growing evidence now further suggests that these two regulative domains are fundamentally interrelated [9]. Dietary lipids can remodel microbial populations that, in turn, generate endocannabinoid-like metabolites that are able to activate eCB signaling pathways. Cannabinoids, on the other hand, can modify endogenous microbiota populations, induce greater levels of SCFAs, and affect systemic immune profiles [10,11]. The generation of microbiota metabolites like SCFAs and bile acids affects eCB receptor efficacy and ligand engagement in both central and peripheral circuits [12].

It seems that the immune system may be the critical intermediary in this triad. Both the microbiota and eCBome have powerful immunoregulatory actions: SCFAs induce the differentiation of regulatory T-cells, suppress pro-inflammatory cytokines, and endocannabinoids modify leukocyte trafficking, cytokine release, and microglial activation. Consequently, perturbations of immune homeostasis may lead to changes in microbial profile and interrupt endocannabinoid signaling [13]. Together, these findings indicate a feedback loop of interconnectedness among microbial ecology, endocannabinoid tone, and immune homeostasis. A fission of this loop may provide a point of commensurable vulnerability across many neurodegenerative and systemic disorders. In order to grossly generalize these converging data, the Endocannabinoid–Microbiota–Neuroimmune Super-System (EMN-S) is proposed [14]. The EMN-S is a conceptual framework for the integration of three functionally different regulatory entities. The EMN-S loop refers to the bidirectional, three-fold feedback between microbiota, endocannabinoid signaling, and immune pathways. From this point of view, the concept of disease may be represented as a loss of EMN-S integrity, wherein feedback fission is manifested as chronic inflammation, synaptic extermination, and systemic failure. Recently conducted multi-omic and clinical studies lend credence to the idea of integrating the EMN-S fingerprint, that is, the person-specific markers incorporating microbial metabolites, endocannabinoid mediators, and immune markers, for the application of precision-based biobehavioral interventions [15].

The import of this integrated model is particularly relevant in the light of the increasing burden of disease manifest as either neurological or psychiatric, since these together constitute more than 25% of disability-adjusted years across the globe (YLD). Alzheimer’s disease, Parkinson’s disease, major depressive disorder, and schizophrenia form one of the more debilitating diseases across the world. Additionally, the prevalence of metabolic syndrome and chronic inflammation increases unhindered, while their interrelationships with neuropathology or psychiatric processes are not well understood [16]. The EMN-S concept gives a unified explanation of these overlapping pathologies, not as concurrent comorbidities, but as manifestations of a common regulatory fission across the microbiota-cannabinoid-immune triad. This position promotes compliance with the principles of systems biology, where the EMN-S is viewed as a network-of-networks, or the latent connectome of resiliency, whereby microbial ecosystems, lipid mediators, and immune circuits are interlocked into dependent functions of one adaptive system. When the integrated frequency of the adapted layers fails, pathological states develop [17].

The objective of this review is to bring forward recent research to support the EMN-S model, evaluate its biological framework, and discuss its implications for disease and treatment.

## 2. The Gut Microbiota as a Neuro-Endocrine Meta-Organ

The human gut microbiome—home to trillions of life forms harboring a collective genome immensely exceeding that of the host—represents one of the most complex and influential regulators of human physiology. Functioning as a neuro-endocrine meta-organ beyond its digestive function, it constantly generates bioactive metabolites, regulates immunologic defenses, and enhances the functional activity of neural circuits. Its composition is most dynamic, changing with diet, lifestyle, and the presence of disease comorbidities, with effects that stretch from early brain development to aging and neurodegeneration [18]. In the past two years, breakthroughs in metabolomics, transcriptomics, and mechanistic experimentation have elucidated how microbiotic metabolites, neurotransmitters, and structural pathways interact to regulate synaptic plasticity, immune tone, and systemic resilience or vulnerability to neurologic disorders [19].

### 2.1. Short-Chain Fatty Acids: Microbial Fermentation as an Epigenetic Code

**Figure 1:**
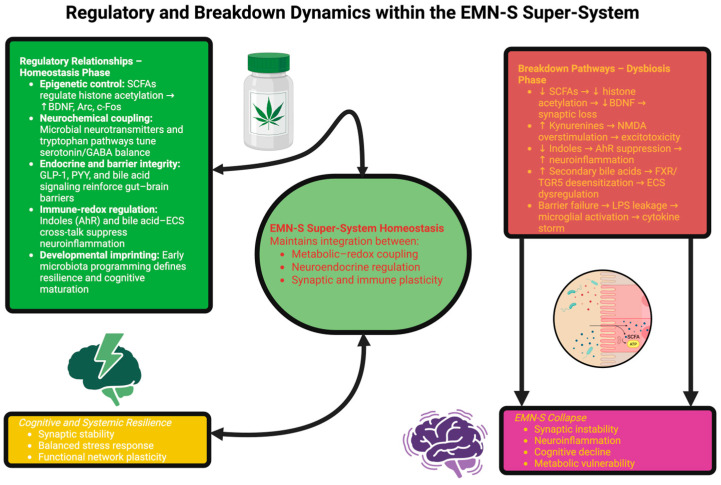
Regulatory and breakdown dynamics within the EMN-S. The diagram shows how the microbial metabolite (SCFAs, tryptophan derivative, bile acids, and microbe-derived neurotransmitter) coordinates and links epigenetic, neurochemical, neuro-endocrine, and immune-redox mechanisms for maintaining the homeostasis of the EMN-S. On the left side, under the balance conditions, the signaling pathways converge to maintain the synaptic plasticity, barrier function, and neuroendocrine homeostasis, which promote cognitive and systemic resistance. However, on the right side, dysbiosis disrupts the homeostasis of the system; loss of the signaling through SCFAs, changed metabolism of tryptophan, increased levels of secondary bile acids, and loss of barrier functions lead to a collapse of the EMN-S caused by the neuro-inflammation, excitotoxicity, and metabolic susceptibility. The arrows show the bidirectional transition from the adaptive state of the microbiome to the maladaptive state that can regulate brain-body communication.

Among microbiotic products, short-chain fatty acids (SCFAs)—acetate, propionate, and butyrate—provide the most explicit example of metabolites functioning in systemic signal capacities. SCFAs use G-coupled receptors (GPR41, GPR43, GPR109A), cross the blood–brain barrier as monocarboxylates, and act as epigenetic modifiers in inhibiting histone deacetylases [20]. High-definition chromatin profiling of neurons and glia exposed to physiologic ranges of SCFAs shows increased histone propionylation (H3K18pr, H3K23pr) and butyrylation, and in association, the up-regulation of plasticity-inducing genes such as BDNF, Arc, and c-Fos [21]. These data provide the mechanistic link between colonic fermentation and transcriptional modulation in the brain. Functionally, SCFAs thus enhance long-term hippocampal potentiation and recover neural performance deranged by dysbiosis or antibiotics. They further enhance microglial maturation, oligodendrocyte differentiation, and white matter integrity [22]. At the level of barriers, SCFAs reinforce intercellular junctions of gut and blood–brain barriers, limiting lipopolysaccharide (LPS) transport and infiltration of neuroinflammation. More broadly, SCFAs act as metabolic substrates, epigenetic signals, and immune mediators, rendering the fermentation of the microbiota a key determinant of neuronal resilience [23]. The schematic below (Figure 1) conceptualizes these multidimensional interactions, highlighting the regulatory pathways that maintain EMN-S homeostasis and the breakdown mechanisms that drive its collapse in disease.

### 2.2. Tryptophan Metabolism: Divergent Pathways into Mood, Immunity, and Excitotoxicity

The metabolism of the essential amino acid tryptophan constitutes yet another signaling pathway driven by microbiota with profound neurobiological consequences. Tryptophan can be funneled into three major metabolic pathways: to serotonin, indoles, and kynurenines, each with specific effects on brain function. Microbiota such as Clostridia sporogenes and Enterococci direct enterochromaffin cells to produce around 90% of systemic serotonin, with important effects on mood and cortical excitability [24,25]. Germ-free mice that are colonized with tryptophan-metabolizing strains of the microbiota exhibit increased prefrontal serotonin activity and restoration of social behavior, providing causal evidence of microbial influence by this mechanism [26].

Derivatives of indole, such as indole-3-propionic acid and indole-3-lactic acid, activate the aryl hydrocarbon receptor (AhR) of astrocytes and microglia. In models of Alzheimer’s disease, indole signaling inhibited inflammatory gene expression, decreased gliosis around amyloid plaques, and enhanced astrocytic neurotrophic support [27]. Conversely, dysbiosis leads to the diversion of tryptophan to the kynurenine metabolic pathway, generating neurotoxic metabolites including quinolinic acid. In human studies, increases in fecal kynurenine to tryptophan ratios were observed to associate with hippocampal atrophy and cognitive deterioration in mild cognitive impairment. The microbial metabolism of tryptophan, therefore, divides into protective and deleterious branches: serotonin for mood and cognition, indoles for neuroprotection, and kynurenines for neurodegeneration [28,29].

### 2.3. Bile Acids: Lipid Messengers Shaped by Microbes

The bile acid pool is yet another significant source of microbiome-derived neuroendocrine signaling. Microbes transform mammal-derived primary bile acids into secondary bile acids with dramatically different biological effects. A metabolomic cohort study focused on primary bile acids identified that the secondary bile acids (i.e., deoxycholic acid and lithocholic acid) were in greater abundance, and the primary bile acids (e.g., cholic acid) were inversely less abundant [30]. These were associated with hippocampal atrophy, tau accumulation, and a decline in cognitive testing. In addition, longitudinal baseline bile acid ratios were used to predict future tau deposits using PET imaging, providing evidence of predictive, not merely correlative, mechanisms [31].

Beyond pathology, bile acids are also thought to signal via FXR and TGR5 receptors located in enteric neurons, vascular endothelial cells, and glia/cells that regulate energy metabolism, synapse signaling, and immune tone. The understandings made visible in interventional studies provide insight into therapeutic utility: tauroursodeoxycholic acid (TUDCA) reestablished dendritic spine density, learning, and the loss of primary bile acids accelerated the loss of synapses. Interestingly, even some of the secondary bile acids were capable of stimulating cannabinoid receptor signaling as either partial CB1 effectors or PPARγ effectors [32]. This non-specific connection links microbial lipid metabolism to ECS signaling, suggesting that microbial lipid metabolism is the biological pathway in which the ECS is embedded in the EMN-S [33].

### 2.4. Microbial Neurotransmitters: The Hidden Neurochemical Reservoir

The microbiota not only indirectly impacts host neurotransmitter pools, it also produces neurotransmitters de novo. Lactobacillus and Bifidobacterium produce GABA, Escherichia coli produces dopamine, Enterococcus probiotics produce serotonin, and yeast, like Saccharomyces, produce histamine [2]. These facts have just recently transitioned from descriptive findings to fully characterizing, in functional terms, by 2024, that GABA-releasing Lactobacillus supplementation affected human cognitive reactivity to negative mood, even demonstrating that microbial neurotransmitters may impact affective cognition. Importantly, pediatric metabolomic research evidenced greater fecal GABA/glutamate ratios in children with autism spectrum disorder and measured cortical excitatory–inhibitory balance, respectively [34]. This indicates that the production of microbial neurotransmitters may directly contribute to neurodevelopmental phenotypes. The microbiota, therefore, acts as a neuromodulatory warehouse where the fermentation of metabolic products may influence neurotransmission in the host, conveying further chemical flexibility to the brain–gut axis [35].

### 2.5. Structural Pathways: Vagus, Barriers, and Hormonal Signaling

The gut microbiota interacts with the central nervous system (CNS) not only through metabolites but also through structural pathways. Of these, the vagus nerve is the most direct path. New computational modeling of SCFA-vagal interactions identified a coding system related to the metabolite and demonstrated that different levels of metabolite generated a patterned afferent firing to encode the microbial states as neural representations [36]. The significance of this conduit is illustrated by experimental vagotomy: severing (tearing) this conduit and the dysbiosis-driven shift in stress resilience and affective behavior is abrogated [37].

The second important pathway is barrier function. Dysbiosis disrupts gut epithelial junctions, provoking systemic leakage of microbial products, including LPS, driving pro-inflammatory cytokines, and priming microglia to a neurotoxic phenotype. Regarding the BBB, SCFAs and indoles promote junctional integrity and re-establish microglial homeostasis after antibiotic clearance of microbes, demonstrating that microbial metabolites protect the barriers in the brain [38]. The endocrine intermediaries complete this triad. Metabolites of microbial origin stimulate enteroendocrine cells to produce GLP-1, PYY, and serotonin that signal locally and centrally, binding gut metabolic function to central satiety circuits, stress circuits, and the hypothalamic circuit, which regulates affect. Vagally mediated, barrier, and endocrine pathways have provided access points into the regulatory hierarchy of the brain [39].

### 2.6. Developmental Programming: Lifelong Imprints on the Brain

The colonization of the organism in early life has long-lasting consequences for the development of the brain. Multi-omic studies show that the microbial composition and metabolomic profiles of the neonate predict cognitive and language development later in life, showing that the infant microbiota provides the neurodevelopmental set points [40]. In brain organoid models with microglia, the microbial metabolites modify synaptic pruning and network maturation [41]. Germ-free mice made without exposure to microbial colonization early in life show abnormal social behaviors, exaggerated stress response, defective pruning, and delayed myelination, all subject to reversal with early colonization [42]. Thus, the microbiota is involved not only in shaping adult brain states but also in programming developmental trajectories that determine across the life course individual vulnerability or resilience [43].

### 2.7. Disease-Specific Dysbiosis Patterns

Recent evidence shows distinct dysbiosis signatures of disease that go beyond correlation. In multiple sclerosis, the dysbiosis of decreased SCFA, Faecalibacterium, and Prevotella correlates with impaired Treg induction and increased Th17 activation, culminating in demyelination. In Parkinson’s disease, it has been shown in longitudinal studies that there is depletion of taxa producing SCFA before the onset of specific motor phenomena, followed by an increase in pro-inflammatory Proteobacteria and α-synuclein pathology [44]. In the case of Alzheimer’s disease, the depletion of Bifidobacterium and Akkermansia correlates with dysregulation of bile acid metabolism and reduced microglial activation, while in the case of autism spectrum disorder, the depletion of the order Clostridiales alters GABA/glutamate ratios, establishing a cortical excitatory–inhibitory imbalance. Together, these findings define dysbiosis as a causal perturbation of EMN-S homeostatic stability rather than an epiphenomenon [45].

### 2.8. Causality and Translational Levers

Causation has been discovered using transplant and engineered strains. Fecal microbiota transfer (FMT) from young, exercise-trained mice to aged recipients induces restoration of hippocampal plasticity, dendritic spine density, and memory function. In sharp contrast, FMT from human subjects with mild cognitive impairment induces deficits of glucose uptake and declines in memory in mice [46,47]. Engineered strains of Lactobacillus show dose-dependent effects on mood and synaptic physiology. Nutritional modulation with certain specific lipids changes the blends of microbial communities, restoring the balance between the endocannabinoid-containing mediators and the lipids of the host. These interventions demonstrate that the microbiota is a manipulable biological system, not just a passive correlate of behavior; hence, there are translational possible entry points for precision microbial engineering [48].

### 2.9. Integration into the EMN-S

**Figure 2:**
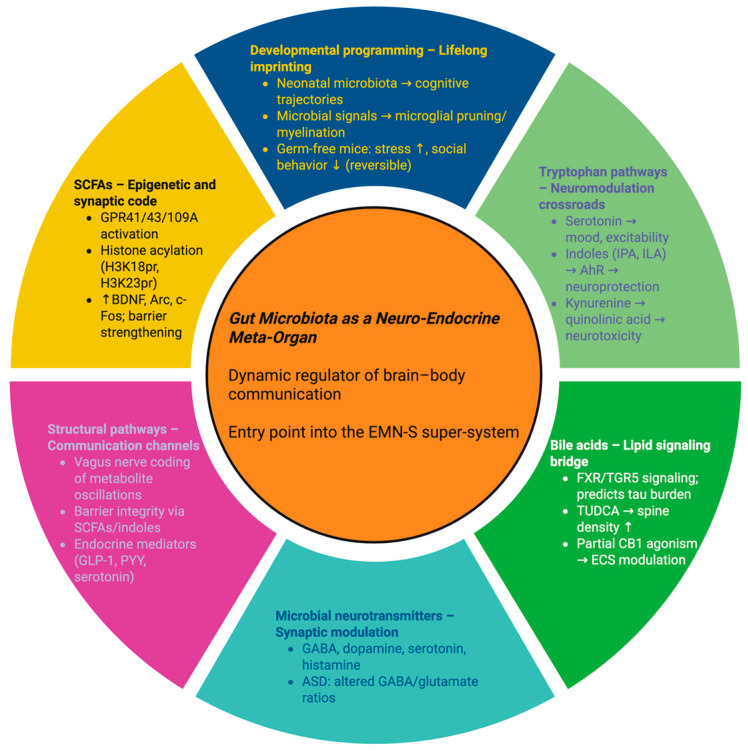
The gut microbiota as a neuro-endocrine meta-organ and entry point into the EMN-S. This schematic is intended to illustrate the principal mechanistic axes through which the gut microbiota communicates with the brain and shapes neural and systemic physiology. Microbial fermentation generates SCFAs that act as epigenetic modifiers, regulate synaptic plasticity, and reinforce barrier integrity. Tryptophan metabolism diverges into serotonergic, indole, and kynurenine pathways, influencing mood, neuroinflammation, and excitotoxicity. Microbial transformation of bile acids engages FXR and TGR5 signaling and connects microbial lipid metabolism to endocannabinoid tone. Microbes also synthesize neurotransmitters such as GABA, dopamine, serotonin, and histamine, contributing directly to synaptic modulation. Structural pathways, including vagal signaling, barrier regulation, and enteroendocrine mediators, provide additional routes of communication. Early-life microbial signals program microglial function, myelination, and synaptic maturation, with effects persisting across the lifespan. Together, these interconnected processes position the gut microbiota as a dynamic regulator of brain–body communication and the metabolic and developmental entry point of the EMN-S framework. Dysbiosis perturbs these pathways, driving inflammatory states and synaptic vulnerability, whereas targeted interventions hold potential to recalibrate EMN-S dynamics and restore resilience.

The evidence described above places the microbiota as the primary entry point to the metabolic and developmental core of the EMN-S. It produces SCFAs that modify chromatin and synaptic plasticity, indoles that recalibrate glial states, and bile acids that shift the endocannabinoid tone, along with neurotransmitters that define cortical circuitry. Structurally, it communicates through the vagus nerve, is involved in maintaining the integrity of barriers, and imprints the neuro-developmental trajectories [49]. Dysbiosis disrupts the action of these mediators, reducing SCFA and indole production, increasing the toxicity of bile acid pools, altering neurotransmitter ratios, and reducing barrier stability, giving rise to an EMN-S collapse. In this state, chronic inflammation, synaptic instability, and systemic vulnerability become evident. On the other hand, targeted microbiota interventions, from nutritional modulation to engineered microbial consortia, may offer the opportunity to reprogram the EMN-S signatures and restore resilience in neurological, psychiatric, and systemic diseases [50,51]. Figure 2 conceptually integrates these neurobiological mechanisms, illustrating how the gut microbiota functions as a neuro-endocrine meta-organ within the framework of the EMN-S. The schematic summarizes the main microbial outputs—SCFAs, tryptophan metabolites, bile acids, and neurotransmitters—as they participate in structural and developmental pathways and are contextualized by the relative dysbiosis destruction of the balance between gut flora in disease. These global interactions define the encoder, integrator, and executor elements of the EMN-S, giving a systems view of how networks of microbes maintain brain and systemic resilience or lead to its destruction.

## 3. The Endocannabinoidome: A Homeostatic Omninet

Initially conceived in the 1990s with the identification of CB1/CB2 receptors and associated endogenous ligands, the ECS had been seen mainly as an odd neuromodulatory circuit. Later work expanded this view to the larger eCBome: a distributed, highly flexible information-transducing network of dozens of lipid mediators, biosynthetic and degradative enzymes, and a large cooperative of both canonical and non-canonical receptors [52]. Although the eCBome should not be thought of as being a linear pathway or being a module limited to the synapse, it is rather a lipidic interface through which neurons, glia, immune systems, vasculature, adipose tissue, and peripheral organ systems are linked. This organized distributive apparatus may be congruent with a context-dependent signaling structure and potent homeostatic function among many internal and external states [53,54].

### 3.1. Canonical Receptors as Adaptive Anchors

CB1, one of the most abundant GPCRs in the brain, is enriched in the cortex, hippocampus, basal ganglia, and cerebellum. In addition to retrograde suppression at sites of neurotransmitter release, CB1 also regulates local protein synthesis in dendritic spines, which mediate the conversion of transitory synaptic events to durable plastic structural and mnemonic changes that link changes in experience to plasticity [55]. CB2, hitherto regarded as biased towards the periphery, is now increasingly understood in microglia and other immune systems to represent a pro-resolving receptor that serves to inhibit phagocytic overdrive, to accelerate amyloid clearance, and to assist transitions from inflammatory to reparative states: CB2 activation in demyelinating models abrogates demyelination, as well as assists remyelination [56]. This functional division of labor—CB1 to enhance synaptic integrity and CB2 to enhance immune tolerance—illustrates a neural-immune dual reach of the eCBome as described in the EMN-S frame [57]. Moreover, the diversity of receptors exhibits, apart from CB1/CB2, further avenues of endocannabinoid signaling. TRP channels (TRPV1, TRPV2, and TRPA1) couple the lipid mediators to nociception, thermoregulation, and excitability. Nuclear receptors (PPARα/PPARγ) couple the lipid signals to transcriptional programs controlling inflammation, mitochondrial biogenesis, and lipid metabolism. The orphan/atypical GPCRs (GPR55, GPR119, and GPR18) carry links to epilepsy and inflammatory hyper-excitability (GPR55), incretin biology and insulin release (GPR119), and tone in blood vessels with traffic of leukocytes (GPR18) [58].

Together, these receptors constitute a polycentric, distributed topographic web as opposed to a hub-and-spoke model, embedding cannabinoid signals in control of pain, metabolic, vascular, and immune networks [59].

### 3.2. Lipid Mediators: The Adaptive Vocabulary

If the receptors constitute the nodes, the lipid mediators constitute the signaling “lexicon”. The canonical mediators anandamide (AEA) and 2-arachidonoylglycerol (2-AG) are complemented by a wider array of N-acylethanolamines (NAEs) and monoacylglycerides. Both AEA and 2-AG are rapidly hydrolyzed by the enzymes fatty acid amide hydrolase (FAAH) and monoacylglycerol lipase (MAGL), respectively, which terminate their signaling activity and tightly regulate endocannabinoid tone [60,61]. 2-AG is abundant in brain tissues, coordinates presynaptic release, shapes astrocyte-mediated Ca^2+^ waves, and contributes to neurovascular coupling—linking neural activity with the dynamics of blood flow [62]. AEA favors stress adaptation and recovery via CB1/CB2, TRPV1, and nuclear targets. OEA and PEA (traditional “entourage” lipids) show anti-inflammatory and anorexigenic actions through the PPARs and are proceeding to clinical testing in pain and neuroinflammation [63]. New mediators such as synaptamide (N-docosahexaenoylethanolamine) in respect of neurogenesis, lysophosphatidylinositol (LPI) as a GPR55 ligand with connections to epileptogenesis, and the N-acyl taurines as modulators of metabolic phenotype show that the chemical vocabulary is wider than that so mapped [64].

Key among the features of this lexicon is that synthesis of lipid mediators is on demand: lipid mediators are made from membrane precursors dependent on the state of the cell, so that situations are signaled rather than tonically, so that rapid accommodation to changes necessary for metabolic, stress, or injury responses occurs [65].

### 3.3. Enzymatic Rheostats and Dynamic Tone

NAPE-PLD and DAGLα/β synthesize NAEs and 2-AG from phospholipid precursors, while FAAH and MAGL degrade AEA and 2-AG, respectively, to turn signals off. Other hydrolases, such as ABHD6 and ABHD12, refine local tone, particularly at neuroimmune and endothelial borders. Disruption to this important layer has considerable clinical importance: the ABHD12 mutations lead to PHARC syndrome, a multisystem neurodegenerative disease following unbalanced eCBome locally [66].

Of considerable importance is the fact that expression of function and activity of the enzymes transductionally is state dependent and may be altered by such factors as nutrient status, circadian phase, athletic or developmental situation, or inflammatory burden. Thus, FAAH has a sleep-waking rhythm tied to cycling, which alters neurotransmitter AEA tone, while expression of MAGL is altered with activation of the immune system. The time-dependent plasticity leads to another time dependence in eCBome control, this being self-tuning over hours and such stages of life as relevant for the homeostasis [67,68].

### 3.4. Systemic Integration Across Domains

Within neural circuits, the retrograde signaling of CB1-dependent signaling alters excitatory–inhibitory ratios, while astroglial lipid signaling modifies neurovascular profusion and glioneuronal communications. Within the immune compartment, the CB2/PPAR pathways lead to downregulation of pro-inflammatory cytokines, increased numbers of regulatory T cells, and resolution of sterile inflammation [69]. Within metabolism, OEA and its relatives influence appetite and levels of energy utilization and mitochondrial function. These systems are critically interdependent. Cortical endocannabinoid signaling produces immune tone. Immune CB2 signaling feeds back on metabolic tone. Metabolic PPAR activation affects the resilience of excitatory neurotransmission [70]. This intersystem coupling means that the eCBome is a systems-level stabilizer mediating transport between plasticity, immune tolerance, and metabolic tone [71].

### 3.5. Evolutionary and Systems Perspective

CB1 homologs are found throughout vertebrates, and lipid-mediated signaling is conserved even in invertebrates, suggesting eCB pathways predated fruiting complex neural systems. From this perspective, therefore, the eCBome is an ancient adaptive tool set later integrated into neural, immune, and metabolic physiology to allow buffering against environmental stressors. Its current function as a disordered lipid-based network is a natural consequence of this evolutionary function at the level of a fabric of resilience across multiple tissue systems [72]. Although computational analogies may be of interest, the key point is biological. The eCBome functions as an integrator, providing the scaffold for the homeostasis of coordination across organ systems [73].

### 3.6. The eCBome as a Homeostatic Omninet in the EMN-S

Within the EMN-S schema, the eCBome functions as the lipid signaling integrator layer, which translates environmental and physiological perturbations into adaptive changes. Receptor array, adaptive mediator inventory, as well as enzymatic rheostasis, ubiquity spatially in a temporally flexible manner, make it supremely suitable for stabilization at the system level [71]. Conversely, perturbations to any of these parameters can travel through the EMN-S loop, for instance, guess-adaptive lipid arrays or receptor crosstalk failure, or loss of tuning via enzymatic mechanisms. The result is patterns seen in collapse states and inflammation, as well as synaptic catastrophes and a widely inaccurate metabolic adaptation. Timed pharmacologic, lifestyle, or microbiota-related manipulations that aim at redefining tone in the eCBome represent plausible levers to re-stabilize the dynamics of EMN-Ss [74].

In synthesis with more recent studies, Table 1 therefore enumerates a series of contextual findings that span those studies that suggest deviations in the encoders, integrators, and executors of major clinical conditions. The aim is exemplary rather than exhaustive: to indicate how it is that rather different collapse trajectories may ensue from the EMN-S schema, but also to indicate the accepted hotspots that call for prospective consideration and discussion.

## 4. The EMN-S Loop: Tripartite Feedback Control

The recent convergence of microbiota ecology, endocannabinoid signaling, and neuro-immune biology necessitates a rethinking of human physiology as a looped network of networks. We internally name this structure the Endocannabinoid–Microbiota–Neuroimmune Super-System (EMN-S): a recursive feedback system wherein microbial consortia encode environmental state; the endocannabinoidome integrates and redistributes signals; and the neuroimmune system initiates adaptive outputs, which, in turn, remodel microbial composition, lipid tone, and barrier set-points. The EMN-S is not a metaphor but a mechanism, a cybernetic organism-within-the-organism, built on feedback stabilization rather than linear causation [81]. Health is a reflection of the loop’s ability to sustain negative feedback and absorb perturbation, and disease reflects the loop’s drift into positive feedback cascades, resulting in collapse. Importantly, collapse is not a sudden onset but a trajectory and can often be recognized years before symptoms by prodromal drift in microbial encodings, pools of lipid mediators, and immunological thresholds. This section formally describes the EMN-S as a feedback system, describes its layers of time and cells, characterizes the possible modes of collapse, and positions early Alzheimer’s as a canonical example of prodrome systematic [82].

### 4.1. Microbiota as the Environmental Encoder

The gut microbiota acts as the environmental encoder of the EMN-S. Through its population metabolism, it compresses a diverse palette of external inputs—diet, circadian shifts, xenobiotics, stress, and infection—into biochemical profiles. But the outputs are not individual signals but multidimensional encodings: ratios of primary to secondary bile acids, amplitudes of SCFAs, balance of indole derivatives, flux in polyamines, quorum-sensing peptides, and emergent classes like N-acyl amides [83]. The (endocannabinoid) profiles are like ecological telemetry, relaying information about the host’s real-time environmental state to receptors in the gut, vasculature, and CNS. The microbiota is the system’s fastest-responding module, as it can adapt to changing substrates in as little as hours, prototyping host responses before any transcription or structural changes have been detected in host tissues [84]. Essentially, the microbiota is an early warning detection system, as mild/moderate dysbiosis can be detected years prior to the onset of pathology. A clear example is that shifts in microbiota were detected in cognitively normal adults with cerebral amyloid/tau pathology in the prodrome of Alzheimer’s, and even back to the previous sentence, the encoder has to inhibit decline prior to a cognition determining if it exists [9].

### 4.2. The eCBome as the Integrative Backbone

The eCBome is the integrative spine of the EMN-S model. Lipid mediators are translators that render microbial encodings, neural depolarization, and immune cytokine inputs compatible with one another. These lipids are readily synthesized “on demand”, can cross many receptor classes and systems, and are present among different tissues in the body, so lipid mediators are distributed middleware that creates some coherence among heterogeneous actions [85]. Enzymatic rheostats (biosynthetic and degradative enzymes) iteratively function to recalibrate tone, adjust loop gain/neurotransmitter responses/homeostasis, and prevent runaway oscillation. In systems terms, the eCBome is the stability apparatus: that is, it absorbs variation, disperses perturbations from their sources, and creates synchrony in domains that otherwise would naturally separate each other. The novelty does not stem from the identities of the lipids but from the function for architecture: the eCBome is the wiring closet, the biochemical internet linking the ecological input to the immune outputs of the ecologically responsive regulators [86].

### 4.3. The Neuroimmune System as Adaptive Executor

**Figure 3:**
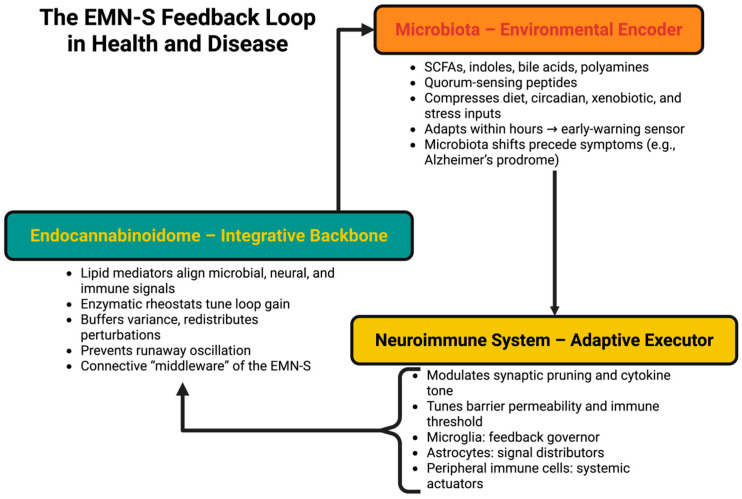
The EMN-S feedback loop in health and disease. The Endocannabinoid–Microbiota–Neuroimmune Super-System (EMN-S) is depicted as a recursive feedback network that encodes environmental inputs, integrates them into coherent physiological signals, and executes adaptive responses across fast, intermediate, and slow timescales. The gut microbiota acts as the encoder, translating diet, circadian cues, xenobiotics, and stress into multidimensional biochemical profiles, including SCFAs, indoles, bile acids, and quorum-sensing peptides. The endocannabinoidome serves as the integrative backbone, aligning microbial, neural, and immune signals through lipid mediators and enzymatic tone control while buffering variance and redistributing perturbations. The neuroimmune system closes the loop as the adaptive executor, regulating synaptic remodeling, cytokine tone, barrier dynamics, and immune thresholds. Disruption of this feedback—through encoder drift, integrator mistuning, or executor overdrive—transforms stabilizing loops into self-reinforcing cascades, driving slow, rapid, or oscillatory collapse. This system view highlights feedback integrity as the substrate of resilience and a target for precision interventions.

Completing the loop, the neuroimmune platform is the executive of adaptation. Microglia, astrocytes, and peripheral immune cells are the coordinators of the changes signaled by microbial and lipid codes: they regulate synaptic pruning, normalize spectra of cytokines, regulate barrier permeability, and set the tolerance threshold. The neuroimmune system is not a passive defender but an optimally functioning governor of baseline excitability, vigilance, and repair [87]. At the level of the cell, microglia are the governors of feedback, mitigating baseline excitability or exacerbating injury and related inflammation; according to the eCBome, astrocytes are the signaling modifiers to relay and amplify lipid and cytokine signaling to neurons and vasculature; and peripheral white blood immune compartments are the systemic actuators that can either facilitate or repress CNS homeostasis [88]. When all of the participants in the system are working together, they inhibit perturbations and restore homeostasis. When the stable code is removed, they are noise suppression and shifted into positive feedback amplifiers, locking the system into inflammatory attractors extending functional disturbances beyond the location of origin [89]. To visualize the 3 components in the context of an integrated system, Figure 3 aims to provide a conceptual representation of how a microbial code, lipid signaling code, and neuroimmune executive function are like links in a feedback system. This conceptual diagram is not intended to make an infographic of everything else; it is intended to illustrate the way these systems become integrated and self-regulating components of a closed-loop system as opposed to linear cascading. In this conceptualization, microbiota are the environmental executive, the eCBome is the coding synapse, and the neuroimmune system is the regulated adaptive executive. To illustrate how a signal in the gut ecosystem is encoded to regulate neural and immune responses, which are altered in turn to alter the microbial ecologies and lipid signaling. This illustrates how the homeostatic parameters of a circuit are maintained with negative feedback (as a stable loop), where coding drift, integrator dysregulation, and executive overadenosine sequence into positive loops that make the disorder.

### 4.4. Feedback Architecture and Temporal Layering

The EMN-S consists of a multi-scale feedback hierarchy. Fast loops (seconds to minutes) function at synaptic and receptor levels, which include lipid pulses that temporarily inhibit neurotransmitter release, attenuate NF-kB activation, or gate calcium dynamics. Intermediate loops (hours to days) involve changing microbial composition, receptor density, and enzyme expression, which reset thresholds for future perturbations. Finally, slow loops (months to years) solidify developmental and degenerative trajectories for long-term changes in connectivity, immune thresholds, and ecological stability [90]. Perturbations cascade upwards; an acute illness or stressor leaves memory (in the form of epigenetic marks) in microbial composition or microglial thresholds, biasing the loop towards vulnerability again—path dependence. In this way, the EMN-S functions as a dynamical system demonstrating critical transitions: as resilience diminishes, variance increases, recovery slows down (i.e., critical slowing down), and the system becomes vulnerable to disintegration [91]. Drawing from ecology and climate sciences, the EMN-S has observable indicators of early warning signals present in increased variability in lipid mediators, reduced circadian amplitude of eCBome oscillations, or increased variance in cytokine tone—all measurable indicators of a tipping point [92].

### 4.5. Collapse States: Dynamics of System Failure

Disintegration arises when the loop breaks free from its iterated recoveries. Here, we use three common taxonomies of collapse:

- Slow collapse (for instance, Alzheimer’s disease), where decades of insidious feedback drift lessen resilience until clinical manifestation occurs [93].
- Rapid collapse (for instance, encephalopathy and sepsis), where overwhelming catastrophic perturbation leads to disintegration of loops in days [94].
- Oscillatory collapse (for instance, relapsing–remitting multiple sclerosis), where the system oscillates between partial (incomplete) recovery and cycled disintegration [95].

A shared substrate of encoder noise, integrator mistuning, or sustainer overdriving feedback loops provides stabilizing feedback to destabilizing feedback loops. Hysteresis is when collapse occurs, the removal of the perturbation does not restore function, and the slow-loop geometry has been recently rewritten. This taxonomy gives disease heterogeneity a promise lexicon: conditions viewed as different could all be seen and framed as different trajectories, as advanced loops failure, or as cyclicality that could be replaced or coded [96].

### 4.6. Early Alzheimer’s Onset as a Systemic Prodrome

Alzheimer’s disease models slow collapse, almost like it is meant to collapse, and ignore it with complete clarity. Preclinical, rather cognitively unremarkable (but amyloid/tau positive) subjects have distinctive microbiota spectra, showing that encoder failed recognitions had already pre-dated the apparent lack of function. Longitudinal shifts in total tau in emergent contextual serum ratios of bile acids in CSF Aβ/tau-PET scans and baseline coordinate true encoder-executor coupling morphometrics deterioration [97]. Likewise, the negative drifts in endocannabinoid tone (e.g., through the loss of anandamides and outrageous dysregulation of CD2) do not somehow inhibit the unintended destabilizing reorientation of microglia. Ultimately, systemic decoupling results in frontal (anterior) meningeal structural outcoupling with executor inertia for temporary inflammatory phases. Such loopy processes, two years before description, reflect systemic prodrome associated with the “detaining phase” of the EMN-S loop decoupling [98]. Ultimately, this systematic view of early-onset Alzheimer’s is, based on the EMN-S loop decoupling, a not fully conceived brief account of protein pathology (not brain pathology). Rather, it is depicted with multi-domain feedback, non-functioning organisms, shared lipid coordination, activation, and immune competence (timing) with each other. Intervening while both are calm, prior to the hysteresis, solidly evokes a positive lock-in, which would clearly be more optimal [99].

- Quantitative Criterial Tiers and Validations of Collapse Dynamics

The critical taxonomical types of slow collapse, rapid collapse, and oscillatory collapse need to be translated into quantifiable biology. This can proceed by the formation of the EMN-S structure in terms of quantitativeness, bilateral criterial tiers involving the correlated evolutions of the three principal axes of the EMN-S, which are the executor (neuroimmune), the integrator (endocannabinoids), and the encoder (microbiota) [100]. Each of these three principal axes evolves its own cadence of drift—anatomical, biochemical, and ecological—and the correlated effluxes of the mean by type of these principles from the mean principally define the geometry of disturbing dynamics of the system [101,102].

- Executor dimension—tau-PET trajectory

The executor inertia is included in the form of the slope of the annualized tau-PET progression (SUVR·year^−1^ (zβτ) standardized). In the condition of collapse, the slope of the system is that of a well-ordered but much milder slope (0.2 ≤ zβτ ≤ 0.6), the variance remaining equal, showing a slow molecular assuaging of the feedback limiting. Rapid collapse here shows a fast theta slope (zβτ ≤ 0.6 (or >90th percentile of controls)), showing an executor overloaded. Reduction in the magnetic decay then shows a swift oscillation in the slope variance with periodic heights in the said 120–360 day bands (normalized power ≥ 0.5), showing an executor in feedback contradicted (cognitively in practice) to his (reactive) other reactions and relapsing dynamics [103]. Within the EMN-S schema, the executor dimension expresses the productive control function of the system: that of the control of the neural-immune feedback that produces proportionate responses to stress [104]. The tau-PET trajectory profile expresses this function on the molecular level: since tau accumulates faster, this expresses functional use of regulatory feedback in the executor feedback loop. A moderate and ordered slope signifies maintained control dynamics; a sharper slope signifies the loss of inhibitory balance and the onset of generalized overdrive. Thus, tau-PET kinetics become an operationally usable measure of the functional integrity of executor feedback and thus bring the EMN-S within the domain of measurable molecular signaling [105].

- Integrator dimension—circadian rhythm variance of AEA

The time-synchronous coherence of the extradited integrator dimension in serum AEA is expressed in terms of the cosinor variance, a parameter derived from 24-h rhythmic modeling using a fitted cosine function. The developments of the contraction of these degenerations will be expressed in terms of a damped oscillation (−1.0 ≤ zA ≤ −0.5; R ≥ 0.6). This shows an ordered restoration. Rapid collapse shows a near-isoarithroidic flat top in its zA ≤ −1.5; R 90th percentile within 12 weeks [106]. Oscillatory collapse shows careful cyclic variances in amplitude (frequencies dominantly of 7–21 days), which thus reflects the slow collapse again of the general cycle. These oscillations in the outer layers systematically break down the chronicled witness of the careful phenotypic differentiations betimes sustained in the biological inertia of the loop in the early superficial collapse of the environmental encoding [107].

The composite classification arises from the mode or common arrangement horizontally with that of the quinquennial dimension. Statistics of the evidence of the patterns thus shown are established with multinomial asymptotics. In the hybrid conditions of one or the other domain of the degenerations of form in continuum, either domain works oscillatory (which exists there together); these meta-modular classifications give information that states unsatisfactory existence is elaborative in part unto the final stage of collapse, which occurs with loss of the generally coherent loop of now irrevocable danger [108].

- Prototypes of validation

These criteria tiers will thus be later developed prospectively throughout and validated by the direct study of the independent longitudinal cohorts (minimum 18-month follow-up) of the overt prodromal neurodegenerative psychopathic affective disorder. These dynamisms will again be encoded specifically by normalized (tau-PET 24 h. AEA) and temporally serially varying SCFA dynamics from continuations by forward operating models of succession of (time-series stresses) lost to the stabilization, or rather to the last of the inherited liberations [109]. The predictive performances will be conducted as if they were vocational, the statistics being performed also about Harrell’s C. and olive leaf (to statistical significance), but also concerning the statistics of the successive cumulative normality and the degrees of calibrational slope obtained with and in respect to the set variances of overdynamics. This secures that in the views of the dimensions of collapse, there comes about a departure from that which is, for the most part, mainly theoretical forms into falsifiable dynamic phenotypes, a satisfactory method of expression in the EMN-S structure, which can now also be analyzed, perturbed, and afterwards normalized [110,111].

This validation phase will extend the assessment of predictive timeliness and cross-domain robustness of these quantitative tiers to a multicenter longitudinal cohort of >500 individuals manifesting with prodromal and early neurodegenerative, affective, and inflammatory states. Individuals will be followed up for a minimum of three years, such that harmonized multi-omic sampling will be performed uniformly across all sites. Blood, stool, and imaging data will be made available at baseline and will be continued every 6 months for the first 2 years of the study, with subsequent annual sampling synchronized with tau-PET, 24-h endocannabinoidomic profiling, and microbiome cuisine and metabolome assay [112,113]. All samples will be taken in the context of an overnight fasting condition (≥10 h) in order to restrict any stray metabolic noise potential to a minimum, and individuals will be subjected to a 48 h washout of any intervention with required medications that would alter either lipid complexion, immune tone, or gut motility pattern [114]. The circadian coherence will be standardized to a fixed zeitgeber (lights on = 07.00), and repeated measures will be anchored to identical clock time across visits in order to control for any differential phase shift masking effects. This design will enable a direct quantification of intra-individual variance trajectories and cross-modal coupling delays and allow time-resolved modeling of loop coherence and onset of loop collapse [115]. By the very virtue of embedding within the study strict pre-analytical control and longitudinal depth, the EMN-S signature will be reframed from a genuinely conceptual constellation of biomarkers to a reproducible, prospectively testable index of loss of resilience across biological and ecological domains [116].

In the fine-tuning of translation fidelity, this exercise in validation must extend past human observations to interspecies calibration [117]. Preclinical dose-response data are harmonized via body surface area conversions to correct for unbound exposure to generate human equivalent dose concentrations, which converge over a range of ±25% of the observed plasma and tissue levels. Blood–brain and blood–gut exposure ratios are to be normalized utilizing *Kp*,*uu* measures to ensure equal parallelism of free fraction penetration in the varying compartments, while measures of CB1/CB2 density and of enzyme expression rheostats (FAAH, MAGL, NAPE-PLD, DAGL) must be corrected for species-specific effects of *Bmax* measured from PET occupancy studies and from single-cell transcriptomic maps [118,119]. Drawings from murine and rat models will then be linked to primate pharmacodynamics and further validated in human cortical–intestinal organoid co-cultures, allowing the dynamic translation of the loop behaviors from the cellular to a systemic level. These adaptations then allow the establishment of a common measure of exposure–occupancy equivalence, which bridges experimental and clinical domains and which ensures that the EMN-S feedback architecture is not merely a species artifact but rather a conserved cybernetic motif measurable across biological hierarchies [120]. To complement these translational calibrations, Table 2 outlines the quantitative framework of EMN-S modulation, summarizing representative compounds—PEA, CB_2_ agonists, and ILA—together with their human-equivalent dose ranges, dosing frequency, exposure windows, and key PK/PD indices.

To find the domain where stabilizing feedback gives way to self-amplifying feedback, we again applied deep phosphoproteomics and single-cell multi-omic methods across microglia, astrocytes, and gut-associated myeloid cells. A single invariant inflection point was traced in what we refer to as the CB_2_–Gi → PI3K/Akt → GSK-3β → NF-κB pathway, a molecular gateway that decides whether neuroimmune signaling remains adaptive or becomes maladaptive [121]. Under conditions of stabilizing feedback, stimulation of the CB_2_–Gi system promotes phosphorylation of Akt(Ser473) and GSK-3β(Ser9) and thus inhibits NF-κB p65(Ser536) activity and maintains negative feedback. During the incipient collapse, loss of the tonic level of CB_2_ stimulation, or the phase misalignment of the eCBome oscillations, produces reciprocal elevations in GSK-3β(Tyr216) phosphorylation, IKKβ(Ser177/181) activation, and opening of the κB-motif, leading to conversion of inhibition into enhancement [122,123].

The phospho-enriched DIA-MS datasets afforded confirmation of the reciprocal Ser9/Tyr216 gating across murine, macaque, and human-derived microglia. Integration of scRNA-seq and ATAC-seq revealed that there was a concurrent upregulation of the cytokines TNF, IL1B, NLRP3, and glycolytic enzymes (HK2, PFKFB3) that occurred with chromatin remodeling toward the increased activation of the inflammatory attractors [124,125]. The same polarity of pattern, observed previously in the glioma growing in the gut-associated macrophage groups receiving the low-amplitude SCFA–indole cycles, was found, in which phosphorylation at TAK1(Thr187) and p38(Thr180/Tyr182) preceded the activation of NF-κB, thus relating the encoder drift phenomenon with that of the executor overload [126].

A minimal switch signature of p-GSK-3β(Ser9)↓, p-GSK-3β(Tyr216)↑, p-RELA(Ser536)↑, p-IKKβ(Ser177/181)↑, plus diminished CB_2_ availability or β-arrestin2 loss was emerging. This molecular fingerprint stands in direct correlation to the value increases and critical slowing in oscillations of cytokines and lipids, heralding here the point of no return, or irreversible positive feedback loop. Phase-aligned remedial intervention with CB_2_ agonists, PEA, and ILA was able to achieve a rapid restoration of the Akt–GSK-3β interactions and elimination of the NF-κB activity, hence restoring the loop synchrony within a time frame of 6–24 h [127,128].

In order to ensure specificity and obliteration of artefactual findings, all polar switch assays were carried out with negative and null response controls. The phosphoproteomic finding of CB2-deficient microglia, together with the complete absence of the Gi pathway, indicated that there was no reciprocal Ser9/Tyr216 transition of GSK-3β and, at the same time, confirmed that NF-κB p65(Ser536) phosphorylation remained unchanged in terms of cytokine challenge, thus making it clear that the transition required the CB_2_/Gi interface [129]. In the dose–response experiment with PEA, exposure to 1.5 µM, greater than, for example, (≈≥1200 mg day^−1^ oral equivalent), caused no further increase in the phosphorylative response of Akt(Ser473) nor reduced variability, indicating a plateau and an unsensitization threshold for the receptor [130]. An objective application of the CB_2_ agonist again failed to reset executor dynamics or achieve the cytokine–lipid synchrony without integrator entrainment, thus indicating the necessity for encoders–integrators co-activation for stable recovery of loops of function [131].

### 4.7. EMN-S Signatures: Predictive Biomarkers of Collapse

Since collapse describes a failure in integration, biomarkers of collapse are multi-domain entities. We use the term EMN-S signatures to denote system-level fingerprints, which comprise inputs from microbial taxa, metabolite ratios, lipid mediator pools, cytokine panels, barrier integrity indices, and neuroimaging readouts. EMN-S signatures are not descriptors; they are predictors—system signatures may diverge long before clinical conversion [132]. There are already exemplars: in the case of Parkinson’s, loss of SCFA producers years prior to the first symptom; in Alzheimer’s, bile acid ratios predicting tau protein dispersion; and in depression, low levels of anandamide predicting stress vulnerability. When combined, concatenating these axes generates a signature space that targets ease of pre-symptomatic stratification, risk prediction, and saliency targeting. In this regard, revolutionizing biomarkers is not about “getting it right” with regard to the molecule but more so about measuring the assimilative capacity of the system [133].

### 4.8. Precision Feedback Medicine

Therapeutics cannot randomly target nodes of a system; they need loop recalibration targets. Thus, we introduce precision feedback medicine: solutions that necessitate restoring negative feedback dynamics across modules. Examples could include re-engineering microbiota (synthetic consortia generating indoles or NAEs), recalibrating the integrator (dietary lipid altering or biased enzyme inhibitors or receptor-specific agonists), and tonifying the executor (microglial-state reprogrammers, rebalancing inflammatory mediator treatment) [134]. Importantly, therapeutics need to reorient to target recovery of the signature, i.e., recovery of circadian amplitude, reduced variance, re-stabilizing mediator ratios, etc., not the specifics of the endpoints. That is, combinations targeting the encoder and integrator may be better than the single node regarding systems with respect to coherence. This means therapy has shifted from reactive correction of later-stage pathology to proactively engineering resilience [135].

### 4.9. Evolutionary Origins and Cross-Species Continuity

The structure of the EMN-S represents significant evolutionary strata of continuity. Lipid signaling predates the vertebrate nervous systems and our microbes, and the chemical genome of the host forms an index in deep time; the crosstalk of the neuroimmune is indexed in all metazoans. The EMN-S is an evolved ancestral survival network that offers a variegated pathway for both static life histories and shifting ecologies. Today’s collapse syndromes—neurodegenerative buffers, psychiatric drift, and autoimmune blunts—may reflect emergent mismatched pathologies, wherein certain ecologies of biodiversity, ecologies of processed food, ecologies of antibiotics, and ecologies of chronic stress destabilize systems that were engineered for resilience in distinct contexts [136]. This, of course, from an evolutionary perspective, reflects that collapse is not distant but a mismatch from the context, and recalibrating may be as simple as recalibrating modern medicine to ancient design specifications [137].

### 4.10. Conceptual Implications and Paradigm Shift

The EMN-S metric provides (rather than health being the index of feedback resistance, disease being the index of the collapse dynamics). It combines and utilizes the language—Loop, Collapse States, Signatures, Systemic Prodrome, and Precision Feedback Medicine—to bring together disparate studies into a single place. Falsifiable prediction: (i) variance and critical slowing in lipid/cytokine oscillations precede disease; (ii) loss of synchrony between the encoder and the integrator portends the prodrome; (iii) two-point interventions are more stable than single-node interventions [138]. Conceptualizes early-stage Alzheimer’s as perceptible and preventable drift—rather than inevitable degeneration. And it sets up a new intellectual architecture: physiology as a cybernetic system, microbiota, lipid signaling, and immune loops constitute one bodily homeostatic organism within an organism. This formalization of an EMN-S loop generates explanatory parsimony and translational potential and establishes the next phase in the resilience therapeutics era: the systems neurobiology research era [139].

## 5. Disease Translation: EMN-S Collapse Trajectories Across Disorders

The EMN-S framework provides value because it views disease through the lens of feedback failure. Rather than categorize disorders based on organ, symptom, or histopathology, the EMN-S maps them onto a spectrum of collapse trajectories—by how the encoder (microbiota) drifts, how the integrator (endocannabinoidome) mistunes, and how the executor (neuroimmune system) either stabilizes or exacerbates perturbations. This shift enables a continuum of collapse states that can be characterized as slow, rapid, oscillatory, developmental, and integrator-drift, explains prodromal signatures well in advance of clinical onset, and provides a translational trajectory toward precision feedback medicine.

### 5.1. Neurodegeneration as Slow Collapse

#### 5.1.1. Alzheimer’s Disease: Systemic Prodrome and Early-Onset Depth

Alzheimer’s disease is the quintessential slow collapse where decades of minor feedback decline precede clinical decline.

At the encoder level, large-scale studies show that serum bile acid (BA) panels are not only associated with cerebrospinal fluid (CSF) Aβ/tau but can predict longitudinal tau-PET trajectories in cognitively normal, MCI, and AD groups. This firmly situates the peripheral metabolic condition as a forward-dominant predictor of executor pathology. The microbiome-based indole-3-lactic acid (ILA) process adds mechanistic causation: supplementation of ILA re-establishes the core AhR microglial–astrocytic tunes (explained through perfusion studies in 5xFAD mice), lowers soluble Aβ, and cognitively enhances cognition, demonstrating that reinstituting a single loss encoder metabolite can strum executor states [140]. At the level of the integrator, preliminary investigations reveal AEA depletion and CB2 dysregulation during prodrome. When run together with BA profiles and tau-PET slope, this provides a tridimensional signature (BA ratio ↑, AEA ↓, tau velocity ↑) that follows the trajectory of collapse with a very high predictive score. At the executor level, tau buildup trajectories are the closest proxy of feedback failure. Tau-PET studies have shown that global tau severity—not amyloid—was the most accurate correlate of cognitive decline, and tau executor kinetics were vastly different within young-onset versus late-onset phenotypes [141]. More than that, microglia–astrocyte cross-talk at plaques can exacerbate pathology irrespective of plaque load. This is an example of an executor circuit that will inherently function as a runaway amplifier once stabilizing encodings are engaged [142].

Early-onset AD (EOAD) provides an extremely refined illustration of collapse chronometry. Autosomal-dominant mutations (PSEN1, PSEN2, and APP) serve as genetically encoded integrators varying γ-secretase processing, wherein the age of onset is advanced by decades. Now, global sequencing of >500 of those variants allows genotype-to-gain mapping, which engenders a positive outlook for patient-specific collapse models. CSF proteomics of presymptomatic carriers showed synaptic, immune, and metabolic drifts a long time in advance of any identifiable symptom, whereby early prodromal biomarker timing is implicated with quantifiable integrator and executor pre-activations [143]. With Down syndrome (APP triplication), natural history cohorts have generated a stereotypical amyloid → tau → clinical succession of collapse as a “genetically modulated” model of gradual collapse [144]. Clinically, EOAD can present with posterior cortical atrophy, logopenic aphasia, or frontal variants, symbolizing each executor’s attractor encoders with integrator drift owing to shared health gain; however, regional network gains distorted their clinically anticipated mapping. Posterior cortex variants show how young brains preferentially collapse in dorsal attention and visuospatial networks, reinitializing their prodrome from memory-oriented to visuospatial/language decline [145].

EOAD signature panel: serum BA ratios (e.g., DCA/LCA conjugates, taurine conjugation index) + circadian eCBome rhythms (Ae. AEA/2-AG peak, PEA/OEA peak levels) + cytokine triad (e.g., IL-6, TNF-α, IL-10) + global tau severity or regional tau-PET slope. These signatures are measured not as static presences in time but as variance contraction, rebound amplitude, and slowed tau buildup, respectively [146]. Conceptual novelty: EOAD is positioned away from “fasted LOAD” or “fast” deteriorations that are derived from LOAD, but rather a slowly collapsing genetically primed and regionalized drift, which can be monitored for years prior to functional decline with multi-domain signatures and treated once EOAD occurs with dual-target interventions [147].

#### 5.1.2. Parkinson’s Disease: Gut-First Prodrome

Parkinson’s disease represents a gut-first collapse in which the loss of SCFA-generating taxa and enteric barrier drift came before dopaminergic neurodegeneration. Prodromal cohorts that are presented with constipation and REM-sleep behavior disorder show encoder drift already and failure in integrator buffering and microglial executor overdrive downstream. From this viewpoint, α-synuclein aggregation is a secondary executor event that occurs as a result of resting the loop, realizing that the executor is not able to be responsibly programmed to escape the fault of failure [148].

PD signature: microbial SCFA indices + 24 h endocannabinoid system (eCBome) oscillations (especially 2-arachidonoylglycerol or 2-AG amplitude) + glial positron emission tomography (PET)/magnetic resonance spectroscopy (MRS) markers of executor tone, assessed for critical slowing in resilience after a change event from stress or trauma [149].

#### 5.1.3. Multiple Sclerosis: Oscillatory Collapse

Relapsing–remitting multiple sclerosis maps onto oscillatory collapse. Encoder states of drivers drift cyclically with seasons; integrator tone drifts; executor boundary drifts from Th17-driven inflammation to tolerance. Collapse does not derive from linear collapse but cyclic instability, causing the cyclical recurrence of symptoms [150].

Systemic inflammatory response is constituted by fecal telemetry, circulating PEA or OEA, and bursts of inflammatory cytokines, analyzed for the amplitude and damping of seasonal oscillations instead of absolute order dynamics [151].

### 5.2. Psychiatric Syndromes as Integrator-Centered Collapse

#### 5.2.1. Major Depression

As a demonstration, major depression is associated with an integrator drift. Multiple studies show reduced AEA and 2-AG (in serum and hair biomarkers), with increased cytokine reactivity. The result is a low-amplitude positive feedback loop: slight encoder variability (diet, circadian disruption) drives executor inflammation that circles back into mood circuits [152].

Depressive signature: hair of plasma AEA; circadian 2-AG amplitude, cytokine triad, pre-post variance, amplitude loss, recovery half-life under standardized perturbations (e.g., sleep restriction) [103].

#### 5.2.2. Anxiety

Anxiety disorders will have an integrator of high gain and poor damping: minimal encodings present an exaggerated executor response.

Note: The signature will be any phase incoherency and excessive variance of eCBome oscillations, which means they are all normal [153].

#### 5.2.3. Autism Spectrum Disorder

ASD exemplifies the developmental constraint of loop attractors. Initial microbial neurotransmitter dysregulation (e.g., raised GABA/glutamate) and illicit/unidentified CB1/CB2 expression trajectories lead the integrator-executor pair to be hyper-excitable and low immune-tolerant. Once established, the thresholds create a stable but atypical attractor state [154].

### 5.3. Systemic Disorders as Whole-Body Collapse

#### 5.3.1. Obesity and Metabolic Syndrome

Obesity serves as a whole-body metabolic collapse, dysbiotic encoders, poor integrator tone (NAEs, 2-AG), and executor inflammation, combined as an adipose-brain attractor. Loss of CB1 availability in brain circuits directly corresponds to cognitive impairment (metabolic collapse enters into cortical control) [155].

#### 5.3.2. Inflammatory Bowel Disease

IBD is executor-dominated collapse: dysbiotic encoder and dysfunctional eCBome buffering result in mucosal immune cell activation all the time. Colon biopsy research shows no CB1/CB2 adherence despite elevated endocannabinoids, a sign of executor amplification despite integrator waiting [156].

#### 5.3.3. Sepsis-Associated Encephalopathy

Sepsis condenses the collapse to days, barrier failure, cytokine storm, and acute endocannabinoid depletion. Prognostic research elevates serum NSE as an executable marker of damage indicators. In EMN-S terms, recovery relates to the return of negative feedback and is considered to look like variance contraction and recovery of synchronous cytokine/eCBome dynamics [157].

### 5.4. Collapse Taxonomy Across Disorders

- Prolonged collapse: AD, PD (decades of drift, prodromal signatures; hysteresis) [158].
- Oscillatory collapse: MS (alternating gain and partial reconsolidation) [159].
- Fast collapse: Sepsis-associated encephalopathy (catastrophic breakdown within days) [160].
- Developmental setting: ASD (thresholds established early—stabilizing atypical attractors) [161].
- Integrator drift: Depression, anxiety (chronic low-grade amplification due to buffering loss) [162].

This taxonomy distances disease in feedback dynamics and bridges neurology, psychology, and immunology.

### 5.5. Translational Implications: From Nodes to Feedback

The EMN-S calls for a treatment evolution from individual biomarker measures to multidomain profiles and node-targeting to feedback occupancy. Instead of running trials on the basis of a single diagnosis, e.g., AD, or prediagnosis conditions, trials will focus on composite signatures (e.g., BA-eCBome-tau in EOAD; SCFA-2-AG-glia PET in PD) by assaying the fixation of loops: reducing variance; circadian amplitude renewal; phase coherence; executor slowing; and not just one or the other within a composite signature cross-identified feedback loop [163].

Under EOAD conditions, such trials would virtually assess two modules, encoder repair (indole-producing synbiotics, bile acid diets) + integrator recovery (CB2 agonists, PEA/OEA nutraceuticals), with assessed composite signature outcomes based on stabilization (in negative feedback) loops, not just or either of those interventions.

### 5.6. Evolutionary and Public-Health Framing

The EMN-S represents a survival loop—the microbial sensing, lipid signaling, and immune tuning happened long before vertebrate brains, and modern derangements/antibiotics/stressors/longevity precariously destabilized it into pure collapse states, potentially missing from ancestral environments. Public health should be a modeled approach to feedback interventions/routines on a population scale—restore circadian rhythms, microbial biodiversity, and lipid tuning to minimize variance, restore amplitude, and attain synchronization [164].

## 6. Therapeutic Horizons: Engineering EMN-S Resilience

In the EMN-S model, pathology represents an unstable state within a feedback loop rather than a damaged component of the loop. As such, recovery necessitates resetting the state of the loop’s dynamics to restore stability, synchronization, and resilience between the encoder (the microbiota), the integrator (the eCBome), and the executor (the neuroimmune system). This perspective aligns with the principles of precision feedback medicine, which focuses on reducing variability, restoring circadian amplitude, and establishing phase coherence between modules to prevent loop collapse [15]. The therapeutic strategies listed below should be regarded as coordinated strategies designed to enhance the encoder’s ability to encode continuously, the integrator’s ability to integrate continuously, and the adapter’s ability to adapt continuously.

### 6.1. Recalibrating the Encoder: Microbiota as the Therapeutic Sensor

Encoder-directed therapies aim to re-engineer the flow of information encoded by the microbiota rather than adding adjunctive effects. While traditional probiotics have been used therapeutically, newer consortia-based designs seek to create well-defined metabolites that can stabilize physiological homeostasis [165]. For instance, certain SCFA-producing microbiota support cortical tolerance and immune tolerance. Indole-producing bacteria (e.g., those producing indole-3-lactic acid) can induce glial cell activation through AhR-mediated pathways, reduce soluble Aβ, and improve cognitive function in amyloid models. Altering the microbial community structure based on diet-responsive guilds (taxa that respond to polyphenols and fibers) can also alter the balance of bile acids away from neurotoxic conjugates toward those associated with greater resilience. Taurine supplementation can redirect the conjugation pathways of microbes to protect against hippocampal injury [166]. Feeding schedules regulate the rhythmicity of the microbiota as much as does the composition of macronutrients. Synchronizing feeding with the circadian rhythm of the host aligns the release of metabolic products generated by the microbiota with the eCBome and cytokine cycles, enhancing coherence of the encoder outputs [167]. Additionally, the use of CRISPR to edit the microbiota and phage targeting to eliminate bacterial genera that produce toxic metabolites and select for beneficial ones can transform the gut microbiota into a programmable early warning sensor and metabolite generator for resilience [168].

### 6.2. Retuning the Integrator: The Endocannabinoidome as Systemic Gain Control

As the integrative layer of the loop, the eCBome provides the gain control necessary to buffer disturbances and amplify signals when necessary. However, nonselective approaches to modifying the eCBome (e.g., inhibiting all FAAH) illustrate the potential risk of disrupting homeostatic processes. Selective partial or biased inhibition of FAAH or MAGL can allow for increases in AEA or 2-AG with reduced receptor desensitization and safer dose ranges [7].

Targeting specific receptors, CB2-biased agonists are currently in clinical trials for neuroinflammation, with indications of efficacy through regulation of microglial phenotype and amyloid processing, without inducing CB1-mediated psychotropism. PPARα/PPARγ agonists expand the scope of endocannabinoid signaling to transcriptional programs that restore mitochondrial bioenergetic function and modulate inflammatory tone, providing long-term correction of integrator dysregulation [169]. Nonprescription buffers like PEA and OEA can provide stability to the integrator tone in chronic pain and neuroinflammatory states and potentially limit the progression to collapse. Entrainment to lifestyle patterns (e.g., optimal sleep, seasonal light exposure, regular exercise) can restore the circadian amplitude of endocannabinoid oscillations and synchronize lipid cycling with microbiota and immune cycles. Collectively, the eCBome serves as a “master dial” of stability, determining whether signals generated by the encoder are amplified or damped [130,170,171].

### 6.3. Reprogramming the Executor: Neuroimmune Circuits as Output Shapers

The executor determines whether disturbances are resolved in a proportional manner or whether they escalate into destructive cascades. Modulating microglial activity is a key mechanism of action: TREM2 agonists (e.g., AL002 in Phase II in Alzheimer’s) and CSF1R-targeted agents can convert chronic pro-inflammatory microglia to debris-clearing and synaptically supportive microglia [172]. Experimental astrocyte modulators (e.g., glutamate reuptake inhibitors, gap junction blockers) are intended to limit the spread of excitotoxicity. Systemic modulation of IL-6/TNF-α and enhancement of IL-10 demonstrate that executor recalibration involves proportional adjustment of response magnitude, rather than generalized suppression of immunity [173].

Strengthening barriers (e.g., gut barrier and blood–brain barrier) reduces noise entering the loop at the input stage; strategies increasing butyrate or omega-3 fatty acids strengthen junctional integrity and decrease the likelihood of immune overdrive. In cases of rapid collapse, executive-first strategies are typically required because stabilizing the output dynamics is the highest priority [174].

### 6.4. Restoring Loop Coherence Through Dual- and Triple-Module Therapies

A collapse resulting from module misalignment is unlikely to be corrected by single-node interventions. Therefore, dual-module strategies (e.g., encoder + integrator strategies), such as using consortia of SCFA- and indole-producing bacteria in combination with CB2 agonists or PEA/OEA, can both reduce the noise of inputs and increase the capacity for buffering. Similarly, pairing integrator + executor strategies (e.g., FAAH-sparing augmentation with microglial phenotype modulators) adds gain control and smooths output dynamics [175].

Additionally, triple-module strategies are theoretically possible and include using chrononutrition to modulate the timing of the encoder outputs; coordinating exercise/sleep to entrain the eCBome oscillations; and using targeted cytokine inhibition to dampen the amplifier properties of the executor [176,177]. The general principle of therapeutic intervention is to achieve compatibility and proportionality of modules, rather than maximizing the effect of a single mediator [178].

### 6.5. Therapeutic Windows Across Collapse States

The timing of therapeutic intervention is as important as the choice of therapy. In the early stages of prodromal slow-collapse (e.g., early Alzheimer’s), interventions directed at the encoder (dietary manipulation, engineered consortia) and the integrator (nutraceuticals) may be sufficient, given the relative preservation of plasticity. In symptomatic early-stage disease, combinations of CB2-biased agonists or FAAH-sparing agents with executor modulation may be able to halt disease progression [179]. In extreme collapse (e.g., sepsis-associated encephalopathy), executor-first strategies (cytokine modulation, barrier strengthening) are typically employed as the first line of treatment, followed by attempts to rescue the integrator. In oscillatory collapse (e.g., multiple sclerosis), treatments should be provided in a phase-specific manner: suppress integrator gain and microbial oscillations during relapses, and foster tolerance during periods of remission. Thus, traditional models of treating disease in a “one-size-fits-all” manner will need to be replaced with models of treating disease based upon trajectory [180].

### 6.6. Dynamic Endpoints for Precision Feedback Trials

Rather than focusing solely on static biomarkers, trial design should emphasize dynamic endpoints. Relevant dynamic metrics include reduction in variance of lipid/cytokine oscillations (stability); lengthening of the recovery half-life of host physiological parameters (e.g., following standardized perturbations including meal tests and sleep restriction); stabilization of circadian amplitude of eCBome mediators (synchrony); and alignment of phase relationships between bile acid ratios, lipid cycling, and cytokine oscillations (alignment) [103]. Trials may incorporate traditional outcome measures with composite measures of loop recovery; for example, in prodromal Alzheimer’s, decelerated tau-PET slope and variance contraction of BA-eCBome-cytokine cycling to measure restoration of resilience [181].

### 6.7. AI-Augmented Feedback Engineering and Digital Twins

The dynamical complexity of the EMN-S necessitates computational augmentation. Artificial intelligence can provide clinicians an opportunity to develop a digital twin of a patient: a virtual model using microbiome sequencing, plasma lipidomics and metabolomics, cytokine profile, and neuroimaging. Utilizing those digital twins, computer simulations can recreate collapse trajectories and show early warning signs, like variance inflation and critical slowing time, long before any symptoms [182,183]. Moreover, these models can be trained and eventually utilize signal detection to guide closed-loop therapeutics, in which wearables detect physiological states, biosensors measure microbial metabolites, and ABMs trigger adaptive responses—adaptive responses could include bang timing nutrition, micro-dosing CB2 agonists, or adjusting probiotics in real-time, as signatures drift. The model of medicine transitions from a fixed prescription plan of care to an adaptive control process plan, with clinical practice echoing the feedback signals of the EMN-S itself [184].

### 6.8. Global Health and Evolutionary Reframing

The EMN-S circuit is an ancient survival loop that is optimized for microbial diversity, dietary diversity, and short life spans. The modern world—processed foods, antibiotics, circadian disruption, and prolonged lifespan—unravels this loop, resulting in collapse phenotypes that have not been seen in evolutionary history. This mismatch consideration reinterprets neurodegeneration, psychiatric disorders, and chronic inflammation as emergent diseases of systemic misfit. Encoder and integrated strategies are the only low-cost and scalable strategies, such as high-fiber diets, omega-3 supplementation, chrononutrition, and sleep entrainment [185]. Therefore, precision feedback medicine grants do not just constitute a personalized therapeutic avenue but also constitute a population-level approach to reducing the global burden of dementia, depression, and systemic inflammation. Public health now becomes the work of feedback-reinforced stabilization of public populations, repairable resilience at scale [186].

### 6.9. Future Horizons in Feedback Medicine

The next frontier of the EMN-S intervention will be in cellular systems medicine and biotechnology fusion. CRISPR-microbiome editing will enable accurate manipulation of the microbiota outputs so that encoders can be developed as therapeutic platforms. Synthetic analogs of endocannabinoids, even with biased signaling and selective tuning for feedback, may reevaluate blunt agonists and antagonists [187,188]. Closed-loop platforms with AI-enabled adaptive therapies will deliver real-time therapies, transforming interventions into living algorithms balancing against dynamic features. Organoids and organ-on-chip plans with microbiota, immune cells, and neural tissue will democratize EMN-S collapse and repair models in vitro. Each of these intellectual properties expresses the future of medicine as feedback engineering, the loop, a described target, and a hypothesized target of intervention [189].

Although there has been considerable conceptual momentum in the area of the EMN-S, the clinical application of this research is still in an early stage, where it can be characterized as being at a transition point. While the primary obstacles to the implementation of the EMN-S have thus far been conceptual, they now appear to be largely structural and infrastructure-related [190]. For example, current experimental approaches continue to be hampered by the lack of integrated multi-omics platforms that would allow for the simultaneous measurement of microbial, metabolomic, and neural variables in real-time. Similarly, patient stratification continues to be difficult due to the extreme variability of the human microbiome, making one-size-fits-all therapeutic strategies impractical. Further, the regulatory framework for the use of microbial therapeutics, genetically engineered probiotics, and biofeedback neuro-devices is currently in its infancy and will require new ethical and safety considerations [191]. However, the horizon for EMN-S-based diagnostics is becoming increasingly clear; over the next 3–5 years, it is expected that the first generation of EMN-S-based diagnostic tools will begin to emerge from coordinated and harmonized biobanking efforts, along with clinical trials using AI-based data analysis strategies to transform microbiome-derived signals into biomarkers for either resiliency or collapse. Over the course of the next decade, it is also possible that precision microbial therapeutics and adaptive feedback platforms could develop into “living” medicines—self-adjusting, context-sensitive systems that could restore network homeostasis [192]. Whether or not the realization of such possibilities occurs will depend on the extent to which scientists, regulators, and technologists can collaborate to bridge the various scientific, regulatory, and technical areas related to the development of “feedback” medicine. If successful, feedback medicine will represent the next major clinical paradigm; if unsuccessful, it will remain an elegant concept [193].

### 6.10. Conceptual Implications

The EMN-S lectures on our therapeutic disposition. Alzheimer’s prevention is plaque clearance to slow tau slope, and no longer is with the eCBome/BA synchronicity restoration. Moreover, depression therapy is a modulation that is no longer fortuitous serotonin but shortening the half-life of recovery and fortifying lipid stores. When intervening in sepsis, bedside care was not reactionary survival but ratifying the decay of damping intervention, preventing executor collapse, and going beyond recovery. This dictum shifts medicine from proactive, isolating intervention to systemic resilience engineering. It recommits to now parsing collapse as occurring from the lens of disease as collapse and therapy as feedback restoring function [194]. The EMN-S framework invokes not only explanatory capacity but also heralds the design of the next wave of innovative interventions that offer a paradigm shift from treating pathology to stabilizing physiology, limited by lapses in the routine and process of a looped system. On examination of intervention strategies, we wish to provide aligned summaries of demonstration studies representing recent research demonstrating a variety of intervention strategies that modulate different modules of the EMD-S [195]. Table 3 is not intended to be comprehensive. To provide, from microbiota engineering to lipid modulation to microglial reprogramming. The intention is to offer a summary to consider feedback stabilization with the protection of the encoder, integrated, and executor systems to summarize approaches to stabilize outcomes.

## 7. Predictive Signatures and Biomarker Integration

The critical challenge for the EMN-S framework is to make the constructs measurable in clinically meaningful ways. Traditional biomarkers tend to be limited to two-dimensional, static measures that are domain-specific (e.g., presence of amyloid plaques, levels of a specific cytokine, or levels of a specific microbial taxon). These are helpful but provide a fixed instance of pathology rather than general movement toward resilience. If disease is the gradual loss of feedback stabilizing, then new diagnostics will endeavor to add time as a variable to capture how a system is changing and be construed as pathology [202]. In addition, this reframing means that you may want to design newer biomarkers to be composite, multidimensional, and longitudinal measures aimed at capturing variability, synchrony, and resiliency of the three modules: encoder, integrator, and executor. Hence, to be clear, these are ideas at a prototype stage, and there are many methods that need to be developed to make them routine, clinically integrated measures.

### 7.1. Encoder Biomarkers: Reading the Microbial Language of Stability

At the point of the encoder, what the microbiota has produced metabolically is the universe of metabolic excretes, not solely based on taxonomic membership. Microbiota metabolic outputs, such as short-chain fatty acids, bile acid conjugation patterns, indole derivatives, and N-acyl amides, are a chemical lexicon with coded messages about diets, circadian rhythms, and ecological balance [203]. Already nearing development are longitudinal studies that highlight how these metabolic output encoders can predict downstream pathology: in Alzheimer’s cohorts, ratios of specific serum bile acid ratios were linked to tau accumulation and cognitive declines 2 years later, and in the Parkinson’s prodrome, the loss of SCFA taxa preceded motor or symptom development [31]. These findings are still very preliminary for the most part and limited to particular cohorts. It will require replication in larger, more heterogeneous cohorts first, prior to any sort of clinical utility. The downside of microbial outputs is also very phenotypic to diet, medications, and lifestyle, and is not easily interpreted. Regardless, the upside is that there is promising capacity to potentially, however microscopically small, encode and ultimately allow, potentially, crystal ball predicting of executor pathology [204]. This is possibly going to be clinically actionable, eventually. Novelty is that it is not just a metabolite, but oscillatory health, circadian phase lock, and perturbation recovery delivery status could determine if the encoder of those behaviors is stabilizing or destabilizing the loop. You can quantifiably observe these longitudinal properties longitudinally, and consistently standardized samples stay along a similar path, which is a downstream pathway to continuing on, just a downstream pathway that is needed [205].

### 7.2. Integrator Biomarkers: Lipid Rhythms as Gain Indicators

The endocannabinoidome provides a 2nd mechanistic biomarker, since it is a non-binary marker of not only disease but also gain index. Anandamide, 2-AG, and other related lipids are oscillated in circadian variation and varying time frames in relationship to stress/sleep/feeding. Repeatedly depressed and stress-disordered subjects have low amounts of anandamide, also based on diminished 2-AG oscillators, which are in relationship with low sleep and abnormal metabolic profiles. Before, in the psychological prodrome of Alzheimer’s, a low anandamide ring with toxic bile acid indexes and no absorption was detected. This could be said to have a particular integrator drifting before the executor loads [62].

What is new about this is not to consider these are like fixed values, but to consider them more like waves. Healthy systems oscillate at larger amplitudes, phase lock with microbial encodings, and have faster available disturbance recovery time. A system representative of moving toward collapse demonstrated variance inflation, exhibited a flatline recovery, and has desynchronized [206]. While this is optimistic, these interpretations are limited by non-standardized assays; namely, endocannabinoids are famously labile, as are circadian phase, diet, and specimen handling for assay outcomes. Therefore, integrator-based biomarkers have potential as possible resilience biomarkers; however, a clinical application relies on standardized and validated use across labs and populations [207].

### 7.3. Executor Biomarkers: Immune and Neural Echoes

Executor biomarkers likely have the strongest predictive ability for clinical outcome because they demonstrate how the system ultimately responds. Target cytokine panel, i.e., IL-6, TNF-α, and IL-10, may help gauge tolerance compared to amplification. In septic encephalopathy, the number of variances and time to resolution of cytokines were negatively correlated to outcomes; executor instability can be seen as delayed resolution. For instance, the tau-PET slope of dementia was sensitive to avenues of executor dysregulation; it positively correlated to clinical decline from amyloid pathology load [208]. Temporal neuroinflammatory PET tracers and MR spectroscopy might allow for the dynamic framing of microglial and astrocytic states, respectively. Novelty with framing of the EMN-S is not the biomarkers themselves, but interpretation; tau-PET is not simply a type or form of pathology; tau-PET has value as a rate of velocity, rate of executor expansion; cytokine patterns are not simply inflammatory markers salivating in compatible biological matrices; we think of them as proxy testing of resilience if the systems return to baseline rapidly, or only after some period of time [209]. Currently, there are few kinetic interpretations in the clinic, but they may serve as a conceptual starting place for rethinking what familiar assays mean in the context of feedback control [210].

### 7.4. Composite EMN-S Signatures: Multidomain Fingerprints

There is the greatest promise in using encoder, integrator, and executor biomarkers in composite signatures. In Alzheimer’s prodrome, it may be possible to utilize a signature that combines serum bile acid ratios, circadian amplitude of AEA or PEA, and tau-PET slope with understanding the relevance of temporally linked domains or the extent to which they begin to drift apart. In Parkinson’s prodrome, SCFA indices, 2-AG circadian rhythms, and glial PET activation could be combined to estimate conversion risk. In multiple sclerosis, microbial periodicity, NAE tone, and cytokine burst could generate oscillation signatures for estimating the likelihood of relapse [75].

The composite types of signatures are compelling because they represent convergence: if one domain drifts but the other domains compensate, it can occur, and the system may remain stable, but if we observe variance inflation or loss of synchrony among several domains, then collapse approaches become probable. Simultaneously, the barriers are also large. Synchronizing data collection across stool, serum, and imaging is logistically complex and challenging; it also requires elaborate analytic capabilities to distinguish signals, and validation across populations is not currently present. Therefore, composite signatures are a highly promising direction and not a reality in the clinic, but they generate examples of how EMN-S principles can be realized [211].

### 7.5. Early-Warning Metrics: From Ecology to Medicine

What is also a unique contribution of the EMN-S perspective is bringing early-warning metrics from ecology and systems theory to the clinic. Variance inflation, critical slowing, and loss of synchrony are well-established indicators of impending tipping points in ecosystems and climate models; their application to human health is just developing. Earlier signs of the presence of these systems have already appeared in cytokine profiles preceding sepsis collapse, in delayed lipid replenishing post-stress, and in desynchronized microbial and endocannabinoid rhythms in preclinical Alzheimer’s [212].

These examples are exploratory, of course. Cross-sectional data will never be able to establish predictive validity, and many more large longitudinal and perturbation studies will be needed. Nevertheless, the simple fact that these marks could indicate a near risk of collapse makes them very intriguing, and if confirmed, could allow a transition in healthcare to predicting instability before pathology occurs, when the opportunity for resilience restoration is still possible [213].

### 7.6. AI Integration and Digital Twins

The growing availability of multi-omic data, together with advancements in computational modeling, tells us that AI may have a meaningful role in integrating EMN-S signatures. Digital twins—virtual representations of patients based on microbiome, lipidomic, cytokine, and imaging data—could theoretically model the evolution of feedback loops and thresholds. These types of models may even predict which fixed intervention best captures the lost synchrony in a person. These initiatives are experimental, and it is a long shot for them to ever become patentable [214]. This is because of the need for having large, standardized datasets, verifiable validation, and avoiding overfitting. While they are still formative, they provide a conceptual framework that demonstrates how intricate signatures might be interpreted in a potentially clinically accessible way. One day, real-time data from wearables and biosensors will be stacked on digital twins (virtual representations of individuals), and perhaps, early deviations will elicit adaptive responses. Whether closed-loop therapeutics can even be integrated into this strategy remains to be seen, but the value of development is worth a try [215].

### 7.7. Ethical and Translational Considerations

The ability to identify trajectory collapse well before symptoms raises ethical considerations. Should an individual be informed that their signatures indicate trajectory collapse, and if so, under what conditions? If there is no option for intervention, then the information could lead to a state of near or real distress. Therefore, a biomarker program including available options (dietary, behavioral, or pharmacological) is necessary to enable prediction to lead to effectiveness [216].

Equally as important is the issue of options. Multi-omic and advanced imaging do not come cheap and, except in research settings, are not available to everyday people. If we pursue these biomarkers indiscriminately, they may further lead to the dismantling of already challenging health equity. A good way to strategize is to prioritize lower-fee and scalable markers (diet-critiqued metabolites, wearable-derived circadian timings) with higher-tech, ensuring resilience diagnostics do not transcend out of public health exploration. Finally, the potential knowledge gained through negative findings can be publicly shared. If an intervention has changed a biomarker or increased but not altered the resilience dynamics, this is good data to shape what is movement from what is loop repair [217].

### 7.8. Conceptual Reflections: Toward Resiliomics

With a glance at the future. One can envision a discipline that is conceptualized to quantification—possibly termed, ebulliently, resiliomics. This does not replace the bodily marker sciences but supplements them, focused on factors that follow the outline, not fitness: variance, synchrony, and recovery. For now, this is an abstract idea, and it will take methodological development, replication, and wide validation to become a reality. Even as a concept, resiliomics will benefit from amalgamating the systematized data, challenging scholars to expound beyond individual analytes and collectively, the feedback loop as a common language. In Section 7, it mentions that future biomarkers will not be single-point-in-time markers of damage but real-time indicators of dynamic life. Encoder metabolites, integrator lipids, and output executor biomarkers, in concert and in the context of variance, synchrony, and recovery, can be multifaceted biomarker signatures of feedback health indicators. These biomarker signatures are both speculative and contend along the way with confounding, assay variability, and ethical constructs of feasibility and respectability. They offer a narrow path to diagnosis that is actionable. If ever, if posed delicately, even with sober reminders about limitations and well-being considerations, that will balance equity-wise, then EMN-S biomarkers may someday allow practitioners to not only observe disease but to remodel collapse trajectories using restorative models before they mitigate their obliteration.

## 8. Integrative Systems Models and Control-Theory Frameworks for the EMN-S

The EMN-S model lends itself not only to biological interpretation but also to formalization in the language of dynamical systems. When the microbiota, endocannabinoidome, and neuroimmune circuits are seen as coupled modules within a feedback loop, disease is the emergence of a shift in system stability rather than just a molecular lesion. This frames the possibility to use insights from mathematics, physics, and control theory to explain how resilience is maintained, how a collapse begins, and how intervention can reinstate stability. While this application is still in its infancy, it represents an exciting opportunity where it will be possible to use the same approaches that explore tipping points in ecosystems and power grids, and even climate dynamics, and apply them to the human brain–body, the same [218]. What follows is the discussion of how systems science concepts, such as attractor landscapes, Lyapunov stability, noise-induced transitions, synchrony, controllability, and entropy, may further our understanding of EMN-S dynamics. Conceptual challenges warrant attention and resolution before clinical applications are made.

### 8.1. Attractor Landscapes and Personalized Stability Basins

As discussed in systems theory, networks are often depicted as attractor basins, like landscapes where depth is indicative of resilience, and slope indicates the rate of recovery. Within this framework, a healthy EMN-S would correlate with a deep basin, whereby the oscillations in microbiota, lipids, and immune regulation are dissipated without any long-term perturbation. As resilience diminishes, the depth of the basin diminishes, and the recovery rate diminishes; thus, the system would be susceptible to shifting to other attractors post-collapse [219]. The path to collapse with Alzheimer’s disease, Parkinson’s disease, depression, and sepsis could each be regarded as attractors, but these might represent different trajectories of collapse. Moreover, the point is that these landscapes are likely to be patient-specific. Genetics, microbial ecology, their lifestyle, and age will ultimately matter and determine their compositions in these basins; thus, classrooms of collapse may be remarkably different patient-to-patient [220]. For example, two patients with similar risk may behave completely differently; one stays in a stable basin, and the other progresses to disease. Following collapse, there is hysteresis; there is an extremely, extremely tedious process to restoring the system for it to ever take the prior state—collapse is almost preclusive until then. This is simply why preventative interventions during prodrome, as the earlier-discussed prognostic interventions in stage, have a curiously elevated level of effectiveness compared to a therapeutic intervention in the indirect high-stakes stages, and why personal mapping of stability basin could probably, as part of predictive medicine, lend a high level of efficacy [221].

### 8.2. Lyapunov Stability and Collapse Velocity

Resiliency is not just about recovering, but the question of how quickly one recovers. Mathematically possible and mathematically reliable, essentially determined by Lyapunov stability, which is deterministic; a recovery, or aftermath, to stabilization once upset. An EMN-S will be characterized by a resilient system of travel along the normalized bile acid profiles to reach stabilization after dietary perturbation or travel along the baseline profiles with adaptive endocannabinoid behavior to stabilize post-perturbation. A vulnerable system would be characterized by moving for a long time with oscillating bile acid profiles and a considerably deteriorating Lyapunov exponent [222].

This could crystallize with collapsing velocity. For example, in Alzheimer’s disease, a subsequent prodromal drift to buying dementia could escape for decades; succession would be insignificant. In sepsis, executor overdrive could produce part-modeled multiple resets in days. There is a loss of basins of stability in either case; however, the length of time of the escape basin will depend on placement. If we could characterize it, collapse velocity might be viable as a prognostic in sequences of predicting such disorders of deficient eroticism, and/or immediate stabilization required when provided [223].

### 8.3. Noise-Induced Transitions and Stochastic Vulnerability

Biological systems exist in a noisy world, with microbial populations varying dramatically day-to-day, endocannabinoid levels fluctuating based on stress responses, and cytokine fields pulsing with daily patterns. In a stable loop, noise is buffered. In a vulnerable loop, noise could push the system over thresholds. Noise-induced transitions are already well-studied in ecology and statistical physics, with random perturbations leading an ecosystem (or material) through a transition into a new state. In the EMN-S, this may provide insight as to why some individuals experience abrupt loss of compensation while others do not—in these cases, a minor infection, course of antibiotics, or even just one night of sleep disruption may act as a stochastic trigger to drive a shallow system collapsing to a deep state [224].

This observation suggests reconceptualizing resilience as not only the depth of attractor basins but also the ability to withstand stochastic shocks. Measurement of stochastic vulnerability could potentially be as important as measuring mean states. This is also practical—that perturbation testing, e.g., standardized meal tests, sleep withdrawal, or controlled stress challenges—to test how well the EMN-S absorbs noise [225].

### 8.4. Synchrony, Coupled Oscillators, and Phase Drift

Each module within the EMN-S is driven by rhythmicity: the microbiota feeds on cycling, the endocannabinoidome oscillates on a circadian cycle, and cytokines exhibit daily and seasonal variation. Under healthy conditions, these rhythms are phase-locked, leading to coherence across modules. As the state of collapse approaches, synchrony dissipates (e.g., fluctuating microbiota SCFA release goes out of phase with lipid peak or flattening of cytokine rhythms) [226].

This could be modeled as a system of coupled oscillators, similar to how the field of neuroscience models synchrony in networks of brain regions. Similar to a loss of coherence in neural oscillations signaling risk for future epilepsy or cognitive decline, phase drift across the microbial-lipid-immune axis may serve as a signal of system collapse. Measuring phase coherence may provide us with a mechanistic explanation of the collapse and may serve as a novel diagnostic. Learning enough across scales and over time (multiscale time-series data) is the name of the game—this is a developmental phase still pushing you to the limits of biomedical technology we have [1].

### 8.5. Controllability and Intervention Thresholds

Control theory adds another dimension. The essential question is what input(s) can put our system back into stability. Network controllability will allow you to specify how controllable the system is to stabilize over time. With EMN-S terms, this becomes whether the encoder controls (microbiota-based), the integrator resets (eCBome changes), or the executor changes (immune changes) afford you the best option [227].

Control thresholds warrant special comment. Some prodromal states have very small thresholds (diet component, nutraceutical supplements) before returning to stability. In other states, once hysteresis really sets in, you would be forced to employ deeper, higher “energetic” interventions. This is where timing is perhaps the most powerful of feedback medicine factors, and control thresholds reveal more information than risk [228].

### 8.6. Entropy, Fractals, and Network Topology

More formal metrics could emerge from EMN-S assessments. Entropy metrics (Shannon entropy, approximate entropy) could be employed to characterize the unpredictability of encoders, or lipid signals, which could be informative; at least entropy is understood to gain its value before instability. Fractal dynamics create yet another route towards conceptually understanding normal physiologic rhythms, ranging from the heartbeat to oscillations of the microbiota, which usually demonstrate a fractal regime, and recovery from disease occurs when this loss of self-organization [229]. Similarly, network topology metrics from network science, including degree centrality, modularity, robustness, etc., could be employed to EMN-S interaction diagrams to locate nodes that, if failure were to occur, would disrupt the loop significantly. According to one author, “metrics to describe the interactions of a system in both theoretical and experimental works, which provide a potential toolbox for quantifying stability, departing from the descriptive biomarkers, which are more traditional focus of pre-clinical assessments, to formal metrics from the system” [230].

### 8.7. Hybrid Models and Data Assimilation

There is no model of the EMN-S that can fully capture it. There are mechanistic models, e.g., differential equations, which provide simplicity but not complexity, or machine learning that does not provide simplicity. One option is mixed models to bridge the mechanical and data-driven learning, e.g., models of oscillators could be gated with lipidomic and microbiome data, and the AI models would be tuned to auto-derive the features based upon real-world clinical pathways [231].

With continuous updates, you would have data assimilation (well known from meteorology) in biomedicine. In weather modeling, the models are modified by incorporating new satellite and sensor data. This could nearly be performed, but the models from the microbiome, lipidomic, cytokine, and patient observational data would all be reconciled on the patient observation data, and to access EMN-S simulations with biology. Technically, this is difficult, but it points toward an exciting future of clinical decision-making based on adaptive conceptual and data-driven models of systemic resilience [232].

### 8.8. Cross-Scale Analogies

Cross-disciplinary analogies can be helpful for thinking through EMN-S dynamics. Microbial phase drift is akin to planetary precession; minor momentary deviations compound over time. The monetary variation relates to the financial markets, wherein minimal shocks destabilize an unstable market paradigm of exchanges. Collapse velocity relates to climate tipping points when there are thresholds related to juxtaposed glacier melting versus progressive desertification. Again, this is not a poetic analogy but a reminder that obliteration is a fundamental indicator of all complex systems confronting coordination, characteristics that are governed inherently by similarities across biology, physics, and societies. There are parallels drawn across different neighboring systems that might prove productive to be able to transfer a means by which to analyze each discipline.

### 8.9. Limitations and Open Questions

While this abstract angle is exciting, these frameworks of reference carry a degree of speculative potential. Conversely, biological systems are much more heterogeneous than the types of ecosystems or climate models; the effort to impose some mathematical construct into clinical practice would at best be complicated. The modulating, truncating, or outlaying metric/methodology–variance/synchrony/entropy/fractal complexity/etc. Which will serve best in populations is unknown [233]. Longitudinal datasets are few, and intervention stimulus testing is limited. Additionally, collapse will not always manifest in a gradual deceleration; in many instances, transitions can be spontaneous and stochastic. And finally, who is to say the ethical quandaries that arise with some responsibility—not responsibility to forecast collapsing trajectories—perhaps how would you disclose this information to the patient?

Acknowledging the above uncertainties will not exaggerate what is possible today, yet it facilitates a personal conversation regarding what could potentially occur in the future [234].

### 8.10. Conceptual Reflections

Using the EMN-S, in conjunction with systems theory, looks clinically to reposition health and disease as the issues of stability, control, synchronization, etc. Collapse is no longer expressed simply as an assemblage of impairment but as shallow attractors, Lyapunov stabilization decay, noise-induced transitions, loss of synchronization, and uncontrollability. Control inputs are no longer drugs or diets but aim to deepen basins, fast-track recovery, enhance buffering noise, and restore coherence. While these concepts are not clinically operational, they are fundamentally a legitimate scaffolding from which one could build empiricism. With purposeful development, they can conceivably help medicine transform itself from pathology treatment to measuring and ultimately restoring resilience itself.

## 9. Translational Roadmap: From Concept to Clinic

We have created a new way to think about diseases—as disruptions to a normal feedback system rather than just damaged tissue. However, we cannot simply present a novel conceptual approach and expect immediate translation into the clinics [235]. There are many steps required for translating an innovative concept into a new therapeutic intervention, including the creation of new experimental designs, clinical trials, regulatory interactions, and public health deployment. We propose a four-step roadmap to translate our conceptual innovations into practice:

### 9.1. Step I: Pilot Perturbation Studies

Step I demonstrates that we can measure and quantify resilience through measuring time-varying responses in humans. We will conduct small, tightly controlled perturbations using standardized meal challenges (e.g., high fat, high fiber, mixed meals) to measure bile acid production and endocannabinoid tone over hours; use sleep restriction protocols to determine if the microbial and lipid rhythms are flattened; use mild psychological stressors to determine how quickly the body recovers and re-equilibrates in terms of cannabinoids and cytokines; and use exercise to determine how quickly the body down-regulates inflammatory processes [103,236]. Our analyses will need to transition from examining static concentration levels to analyzing recovery curves, half-lives, variance, and cross-module synchrony. Rapid and proportional recovery is indicative of a resilient system; delayed, desynchronized, or noisy recovery indicates a vulnerable system [163]. Successful demonstration of proof-of-concept in Step I provides evidence that our EMN-S metrics can transition from a theoretical approach to actionable human readouts [237].

### 9.2. Step II: Biomarker Standardization and Infrastructure

Translation of our innovative concepts requires the establishment of a standardized method for assessing these measurements. Circadian rhythm influences endocannabinoid levels; microbial outputs vary based upon diet; cytokine assessments are sensitive to pre-analytical procedures. An international consortium should develop a set of reference conditions (i.e., fasting state, time-of-day windows, plasma endocannabinoids); standardize stool collection and sequencing protocols; and link cytokine panels to calibrated standards [238].

Creation of a shared computational resource is essential for the translation of our concepts, development of validated pipelines for integrated multi-omics/imaging, pre-registration of analysis plans, and linking of repositories for ensuring reproducibility and progressive refinement of metrics across different populations. Without establishing standardization, dynamic endpoints will remain mere curiosities; with it, they will become credible biomarkers for clinicians, regulators, and patients [239].

### 9.3. Step III: Clinical Trials and Dynamic Endpoints

In Step III, our conceptual innovations will be embedded within the design of clinical trials. Static endpoints (e.g., symptom scales, single timepoint biomarkers, event rates) will continue to be used as necessary; however, we will add dynamic endpoints that assess resilience: variance contraction, recovery half-life, restoration of circadian amplitude, module synchrony, and collapse velocity (the rate of pathological progression) [240]. Examples: in the context of prodromal Alzheimer’s disease, engineered synbiotics plus CB2-biased agonists were assessed using both tau-PET slope and re-establishment of rhythmicity in both bile acid and endocannabinoid profiles; in the context of MS relapse prevention, synchronization of microbial–cytokine oscillations; and in the context of depression, reduction in endocannabinoid recovery time post-stress [241].

Regulators will carefully examine the novelty of our proposed dynamic endpoints. A reasonable strategy would be to initially utilize them as secondary endpoints and build predictive validity and sensitivity to treatment effects. Once we establish superiority, we can then promote them to primary endpoints and begin to shift our trials from pathology suppression to restoring resilience [242].

### 9.4. Phase IV: Implementation and Global Integration

To achieve scalable and equitable implementation, we will need to simplify our measures and make accessible technologies available. A minimal panel of clinical measures could include SCFA ratios, bile acid indices, anandamide tone, and compact cytokine triads [243]. Wearable devices can monitor sleep, circadian rhythms, and heart rate variability (HRV); ingestible devices can sample gut metabolites; and biosensors can provide near real-time cytokine/lipid readouts. Cloud computing can integrate data from wearable, ingestible, and biosensor devices to create resilience dashboards; artificial intelligence (AI) can identify early deviations in a patient’s trajectory and suggest adaptive interventions [244].

Then we might think of having “resilience check-ups”—or linking them to cholesterol, blood pressure, or glucose—and giving annual resilience index scores to vary, correlate, and recover. These would not predict collapse deterministically, but they would help stratify risk for prevention and early intervention. We would also require clinician training on resilience check-ups and shifting clinical culture from static or temporal snapshots of biomarkers to dynamic systems interpretation [245]. On a population health scale, resilience monitoring at the public health level is possible. Wastewater metabolomics could measure the microbial signature of resilience at the population level, early tracking neighborhoods at risk of collapse-linked diseases. National health surveys could add resilience scores or measures to reframe nutrition, sleep, and circadian hygiene policies into resilience engineering. This part of the framework demonstrates an opportunity for EMN-S medicine to help to structure not only clinics but also societies [246].

### 9.5. Policy, Precision Medicine, and Preventive Healthcare

Our EMN-S framework aligns with the goals of precision medicine, particularly those focused on integrating multiple omic types, and the goals of preventive neurology, which aim to intervene prior to the onset of symptoms. Our EMN-S biomarkers can assist in the early stratification of individuals with Alzheimer’s disease/Parkinson’s disease and reframe lifestyle medicine (diet, sleep, exercise) as a means to restore feedback stability rather than providing general wellness benefits [247].

Public policy can operationalize resilience through a variety of mechanisms, such as subsidizing fiber-rich food options, enacting regulations that minimize circadian disruption (e.g., shift schedules, urban lighting), and incorporating resilience metrics into public programs. Intersectoral collaboration expands the scope of the evidence base and relevance of the EMN-S across neuroscience, public health, and policy communities [248].

### 9.6. Economic and Societal Impact

Collapse late in life is expensive (dementia care in institutions, ICU stays for sepsis, lifelong immunosuppression). Lifestyle medicine and pharmacological interventions that restore feedback stability are preventive and less costly. Even moderate delays in collapse can produce significant cost savings.

Modeling cost-effectiveness should become a key component of our translational efforts: if our predictive models can forecast Alzheimer’s progression or sepsis outcomes with similar accuracy—or better—than current diagnostic tools, then there will be increased willingness among payers and policymakers to adopt early and less costly interventions [249].

### 9.7. Regulatory and Clinical Adoption Barriers

Challenges are anticipated: regulators will be concerned regarding the use of dynamic endpoints, clinicians may resist moving away from lesion-focused assessments, and patients may be concerned regarding the implications of having predictive labels assigned to their medical history. Strategies to mitigate these concerns include engaging with regulators early in the process, incorporating dynamic endpoints into existing clinical trials, developing clinician training materials with visual aids that clearly illustrate how to interpret resilience metrics, and designing the resilience metrics themselves in ways that emphasize strength-based assessments rather than stigmatizing [250].

Ultimately, adoption will occur when the clinical utility of our metrics is demonstrated to either improve outcome predictions or facilitate more effective treatments [251].

### 9.8. Digital Health Ecosystem

Continuous resilience monitoring is facilitated through the use of digital health technologies. Wearables can continuously monitor sleep, circadian rhythms, and HRV; ingestible devices can continuously collect gut metabolite samples; and biosensors can continuously collect near-real-time cytokine/lipid readouts. These continuous data streams can be synthesized through cloud computing into individualized resilience dashboards, and AI can analyze these data streams to detect early deviations in a patient’s trajectory and suggest adaptive interventions [252].

Digital twins represent a next-generation application of the EMN-S; rather than simply monitoring physiological functions, digital twins can simulate feedback trajectories, evaluate the effectiveness of potential interventions in silico, and guide closed-loop therapy. The successful development of digital twin technology will require addressing issues related to data quality, patient privacy, and clinical acceptance; however, digital twin technology represents a logical extension of the EMN-S from monitoring to adaptive control [253].

### 9.9. Global Equity and the Global South

The translation of our innovative concepts must address issues related to inequities. Many of the conditions associated with collapse are found in low- and middle-income countries, where access to advanced omics and imaging techniques is limited. Therefore, we prioritize the development of low-cost proxies (e.g., stool metabolite strips, structured dietary recalls, and inexpensive circadian rhythm monitoring) and population-level programs to enhance microbiome diversity, increase fiber consumption, and improve sleep hygiene [254].

### 9.10. Conceptual Reflections: A Medicine of Resilience

In Section 9, we placed the EMN-S not only in the realm of laboratories and clinics but in the broader landscape of healthcare, policy, economy, and society. The roadmap of phased translational, resilience “check-ups”, policy integration, economic rationale, regulatory alignment, digital ecosystems, and global equity lays out an integrated path to moving from theory to practice. Each step is an arduous undertaking, each step will require interdisciplinary cooperation, and each step contains unknowns. But together they point in a direction that implies resilience medicine is possible, not a distant ideal but a real-time view of your potentially rich horizon.

With rigor, humility, and inclusiveness, the opportunity to clearly move forward with the EMN-S could fundamentally change not only how we study disease but, more fundamentally, how we organize all healthcare systems—this leads to new social and ethical questions. The EMN-S posits that medicine should not just fix damage (that may or may not occur) but continually check how to strengthen the feedback loops that lead us to health in the first place. In this way, the translational roadmap is a plan for not just a paradigm—it is an opening to completely rethink the core of medicine in its entirety: the science and practice of resilience.

## 10. Conclusions and Future Perspectives

The EMN-S framework provides medicine with an alternative way to view health and disease. Much of modern biomedicine has been defined by the exploration of lesions, a single molecule, or linear causation. The EMN-S focuses on stability dynamics, or the constellation of feedback loops related to microbial encoders, lipid integrators, and neuroimmune executors. Within this view, disease goes from damage to erosion of resilience by losing the appropriate proportion, synchrony, and damping to tolerate stress. This reframing does not negate traditional knowledge but subsumes it, providing a logic where resilience is co-centric with pathology to understand health and disease. In this reinterpretation, the EMN-S proposes a transition from lesion-focused medicine to resilience-focused medicine—a change that is subtle in logic but broad and deep in impact.

To proceed further, the medicine and biology fields must establish several grand challenges. First, resilience must be quantifiable: universal biomarkers of variance, synchrony, and recovery need confirmation in heterogeneous populations. Second, computational infrastructure needs to be established for these signatures themselves to establish patient-specific digital twin models capable of predicting collapse trajectories. Third, resilience assessments must be incorporated in clinical care—what we shorthand call resilience check-ups—for feedback stability to be tracked as accurately as blood pressure or cholesterol levels. Fourth, treatments must be verified, not simply based on symptomatic responsiveness, but in a manner that identifies the ability to gain feedback control, together deepening attractor basins and reducing recovery time. Ultimately, resilience medicine must be equitable, with low-cost proxies and community-scale tools that enable global populations to experience its benefits. Addressing each of these challenges is daunting, but addressing them collectively would convert the EMN-S from a theoretical construct into a clinical construct. Interdisciplinary collaboration is needed for progress to happen. The language of attractors, synchrony, and noise-induced transitions is familiar to physicists, mathematicians, and ecologists; the language of digital twins and adaptive control is shared by engineers and computer scientists; and the language of biomarkers and therapies is in the purview of all clinicians and neuroscientists. The EMN-S could become a crossroads for all the disciplines. Mathematicians could polish the models of collapse dynamics; ecologists could contribute eco-knowledge about tipping point experiences; engineers could create adaptive control systems; and clinicians could see if resilience metrics predict actual outcomes. With representation from diverse sectors, resilience medicine could make strides more effectively and reliably than if it were to proceed in isolation from biomedicine.

Medicine has historically evolved through significant shifts in conceptual paradigms. Germ theory redefined disease as infection, antibiotics made microbial threats manageable, genomics unveiled the molecular building blocks of life, and immunotherapy reframed cancer as an immune problem. These EMN-Ss belong to this process of reframing: we propose the frontier of study and focus is to measure and maintain resilience itself. If germ theory instructed us to find pathogens, resilience medicine may guide us to find steadiness. If genomics has taught us to interpret DNA, resilience medicine may teach us to interpret feedback loops. When housed in historical context, it becomes evident that the EMN-S is not a departure from medical history but rather a way of carrying it forward, the next leg of a long journey of framing. Possible future scenarios are easy to imagine and shocking in their ramifications. By 2040, a typical annual check-up might include, in addition to cholesterol and glucose, a resilience index based on the circadian period of cannabinoids, the synchronicity of microbial metabolites with cytokines, and the half-life recovery from a mild disturbance. Patients might go home carrying their personalized stability maps, illustrating locations of deep and shallow basins of health. Communities could monitor resilience through wastewater metabolomics and circadian monitoring to pinpoint communities facing the risk of dementia epidemics or acute inflammatory storms. Health systems could intervene upstream through nutritional subsidies of fiber-based diets, triaging circadian-disruptive work schedules, or promoting light hygiene, as negative wellness trends, but not any other trends that lack upstream stabilization for feedback. Resilience medicine in this vision shifts from the clinical to communities, from the individual to the societal.

Simultaneously, we must be humble. Collapse is complicated, frequently nonlinear, sometimes abrupt, and not always predictable. Early-warning signals may be imperfect, models may be simplistic, and intervention may not stabilize as we would hope. Predictive and diagnostic assessments need to be ethically distributed, interventions must have global access, and equity must be prioritized. Therefore, we hope the EMN-S is not a terminal doctrine but instead a conceptual invitation—a potential framework for engagement, refinement, disruption, and extension. The EMN-S is useful not in its propositions, but in the research it incites. And yet, if even part of the above envisioned future is true, the depths are profound. Medicine based on resilience and not waiting for collapse could delay/deter the advancement of dementia, alleviate the impacts of infection, prevent recurrences of autoimmune conditions, and diminish the worldwide burden of chronic inflammatory conditions. A medicine grounded in engineered stability could be game-changing to public health efforts focused on promoting resilience as a public good. Perhaps more importantly, medical work is not in overcoming collapse but sustaining coherence—ensuring rhythms, synchrony, and proportions that ultimately constitute human life.

We end not with an ending but with a beginning. The EMN-S is embryonic, its propositions speculative, and its usefulness unsubstantiated. However, it exemplifies our journey activating stability—from harm to stability, from symptoms to feedback, and to chronic illness as resilience. Our hope is that all who engage this framework—clinicians, scientists, and theorists—will authenticate, engage, and iterate on this framework. If medicine is simply maintenance of feedback loops, it also has to be the re-stabilization of resilience, and the EMN-S is offered as a prologue—a stage for an advancing constellation of futures to align—a preamble, not an epilogue.

## Figures and Tables

**Table 1:** Aims to summarize original 2024–2025 findings relevant to the encoder (microbiota–metabolite outputs), integrator (endocannabinoidome mediators), and executor (immune–neural outputs) modules of the EMN-S across representative conditions.

Indication	Encoder Drift (Microbiota/ Metabolites)	Integrator Imbalance (Endocannabinoidome)	Executor Overdrive (Immune/Neural)	EMN-S Collapse Trajectory (Inference)	References
Alzheimer’s disease (AD)	Peripheral bile acid (BA) signatures align with CSF Aβ/tau and PET amyloid/tau, indicating long-horizon encoder drift; microbial aromatic–tryptophan outputs act as stabilizers: indole-3-lactic acid (ILA) reduces Aβ via AhR signaling, and indole-3-propionic acid (IPA) (from *C. sporogenes* synbiotics) improves cognition in AD models.	Human eCB tone drift is emerging in prodromal spectra and plausibly co-moves with lipid/BA axes; paired lipidomics–metabolomics (serum–stool–CSF) is recommended to capture integrator buffering loss.	Tau-PET positivity and slope are the strongest near-term predictors of clinical transition vs. amyloid; ML on large tau-PET cohorts improves risk stratification and subpopulation discovery.	Slow-drift collapse: years-long encoder shifts (BA, indoles) erode integrator homeostasis; executor acceleration is indexed by tau-PET kinetics toward symptomatic conversion.	Nabizadeh et al., 2024; [75]
Parkinson’s disease (PD)	Multiple cohorts show SCFA depletion and microbial pathway reprogramming; spouse-controlled prebiotic pilots (dietary fiber + lactulose) elevate fecal SCFAs and partially normalize metabolomes, supporting encoder rescue feasibility.	CB_2_ upregulation on microglia is seen across neuroinflammatory states; PD-relevant studies and reviews support CB_2_-targeted modulation to attenuate microglial activation and dopaminergic injury—an integrator gain-drift/compensation axis.	Microglial activation and nigral degeneration across temporal windows; glial crosstalk and disease-associated microglia states track progression and inflammatory tone.	Gut-first oscillatory collapse: encoder SCFA loss → integrator CB_2_ tone drift → executor neuroinflammation, producing a motor prodrome years pre-diagnosis; combined encoder–integrator rescue is predicted to damp executor outputs.	Bedarf et al., 2025; [76]
Multiple sclerosis (MS)	Primary BA metabolites at baseline predict slower brain and retinal atrophy; TUDCA RCT in progressive MS is safe and shifts immune and gut features—human interventional anchor for encoder modulation.	Oscillatory N-acylethanolamide/eCBome tone is plausible; BA–lipid crosstalk and circadian sampling are recommended, given serum–CSF lipid decoupling and weak cross-compartment correlations.	CSF lipid modules correlate with cytokines, disability, and MRI activity, offering executor-state readouts that align with metabolomic encodings.	Relapsing–remitting oscillatory collapse: episodic encoder–executor coupling with seasonal or flare-linked imbalances; bile acid augmentation (e.g., TUDCA) is a rational stabilizer candidate.	Ladakis et al., 2025; [77]
Major depressive disorder (MDD)	Diet/sleep and stressors modulate microbiome outputs, but prospective recent human encoder metabolites tied to trajectories remain scarce—priority for perturbation trials with dense sampling.	Endocannabinoid tone: plasma AEA/2-AG alterations replicate; pediatric RCT cohort shows lower hair AEA as a longitudinal marker; in severe MDD, ECT courses track with plasma eCB shifts (and historical CSF AEA rises post-ECT).	Low-grade inflammatory variance intersects with the eCBome; multi-timepoint designs are needed to parse trait vs. state.	Bidirectional drift: stressor-tuned encoder noise + integrator amplitude loss → executor dysregulation; quantify recovery half-life as a target for trials.	Amin et al., 2023; [78]
Sepsis-associated encephalopathy (SAE)	Acute dysbiosis is likely but under-sampled in ICU windows (narrow encoder capture); methodologically, the earliest feasible encodings may be plasma metabolome ± stool if available.	Rapid NAE/eCBome swings are plausible integrator shock responses; prospective ICU lipidomics are needed to define amplitude and timing relative to organ failure.	Serum neuron-specific enolase (NSE) rises in SAE and improves diagnosis/prognosis; a recent cohort shows NSE + cerebral oximetry (rSO_2_) enhances identification and outcome prediction; multiple meta-analyses link higher NSE to mortality and adverse neurological outcomes.	Catastrophic rapid collapse: executor storm dominates early; composite NSE + physiologic monitoring provides an actionable signal while encoder/integrator streams lag.	Zhang et al., 2025; [79]
Inflammatory bowel disease (IBD) (gut–immune EMN-S comparator)	Disease–activity-linked bile acid remodeling is repeatedly observed; multi-omics studies show altered primary vs. secondary BA balance and microbial BA-metabolizing taxa shifts; interventional nutrition/phytochemicals (e.g., berberine) re-align BA–microbiome networks.	Human tissue shows altered CB_1_/CB_2_ expression with nociceptin (NOP) correlation; mucosal eCBs and oxylipins are elevated and track cytokine expression—an integrator remodeling axis in active disease.	Cytokine fields and innate immune cells (e.g., neutrophils) define executor burden and flare dynamics; linking mucosal eCB shifts to systemic immune variance is a near-term translational aim.	Regional oscillatory collapse: gut-centric encoder–integrator remodeling with executor flares; instructive analog for brain-directed EMN-S hypotheses.	Diab et al., 2019; [80]

**Table 2:** Pharmacokinetic, pharmacodynamic, and dosing parameters of EMN-S modulators. Summary of representative agents—PEA, CB_2_ agonists, and ILA—used to recalibrate encoder, integrator, and executor axes of the EMN-S. Values indicate approximate human-equivalent dose ranges, dosing frequency, therapeutic windows, and key PK/PD metrics derived from harmonized preclinical and early translational data. The sequential regimen (ILA → CB_2_ agonist → PEA) defines a 6–24 h composite window restoring feedback coherence and limiting receptor desensitization. Abbreviations: PEA, palmitoylethanolamide; ILA, indole-lactic acid; CB_2_, cannabinoid receptor 2; AhR, aryl hydrocarbon receptor; PPAR-α, peroxisome proliferator-activated receptor-α; RO, receptor occupancy; K_p,uu,brain_, unbound brain-to-plasma ratio; T_1/2_, half-life; C_max_, peak concentration; F, bioavailability; AUC, area under the curve; SCFA, short-chain fatty acid; NF-κB, nuclear factor κB.

Compound/Class	Targeted EMN-S Axis	Mechanistic Role	Dose Range (Human-Equivalent)	Dosing Frequency	Therapeutic Window (Effective Interval)	Combination Sequence/Notes	Key PK Parameters (Approx.)	Preliminary PD Effects
Palmitoylethanolamide (PEA)	Integrator (eCBome)	Endogenous lipid amide; PPAR-α and CB_2_ co-modulator; stabilizes lipid-immune tone and reduces glial excitability	300–1200 mg day^−1^ (oral)	Once or twice daily	6–12 h	May be co-administered within 4 h after CB_2_ agonist or ILA to extend feedback coherence	T_1/2_ = 6–8 h; C_max_ = 0.2–0.5 µM; oral F ≈ 0.4	↓ cytokine variance, ↑ circadian AEA amplitude, restored integrator gain
CB_2_-Selective Agonists (e.g., JWH-133, HU-910, β-caryophyllene)	Executor (Neuroimmune)	Microglial state-reprogrammer; reinforces anti-inflammatory tone and restores executor homeostasis	0.05–0.2 mg kg^−1^ eq. (oral/parenteral)	Every 24–48 h	2–8 h post-dose (peak 2 h)	Administer 12 h after ILA preconditioning; co-administer PEA within 4 h	T_1/2_ = 4–6 h; K_p,uu,brain_ ≈ 0.25–0.35; RO = 50–70%	↓ microglial activation, ↓ NF-κB, ↑ negative-feedback recovery
Indole-Lactic Acid (ILA)	Encoder–Integrator Interface	Microbial AhR agonist; modulates tryptophan metabolism and SCFA coupling	50–150 mg kg^−1^ eq. (oral)	Once daily	3–6 h	Administer 12 h before the CB_2_ agonist to prime microglial sensitivity	T_1/2_ ≈ 3 h; portal C_max_ = 5–15 µM; high first-pass	↑ microbial synchrony, ↑ intestinal barrier tone, entrains circadian microbial-lipid phase
Combined Regimen (ILA + CB_2_ Agonist ± PEA)	Multi-Axis (Encoder + Integrator + Executor)	Sequential loop recalibration restores encoder precision, integrator amplitude, and executor stability	Derived from above	Cyclic protocol: 36 h interval	6–24 h composite	ILA → 12 h → CB_2_ Agonist → 4 h → PEA	Composite AUC match within ±25% across axes; steady-state after 3 cycles	Reduced variance inflation, normalized cross-modal coupling, and increased resilience index

**Table 3:** Intends to summarize therapeutic classes with evidence from recent studies, organized by their primary EMN-S target (encoder, integrator, executor). Each entry specifies mechanism, type of evidence, outcomes, and stage of development, together with an interpretation of the intervention’s potential leverage on feedback stability. The studies included are illustrative rather than comprehensive, and the framework emphasizes how interventions might reduce noise, restore circadian amplitude, or reshape executor responses. Abbreviations: BA, bile acid; CB2, cannabinoid receptor 2; NAE, N-acylethanolamine; OEA, oleoylethanolamide; PEA, palmitoylethanolamide; RCT, randomized controlled trial; TREM2, triggering receptor expressed on myeloid cells 2; TUDCA, tauroursodeoxycholic acid.

Intervention Class	Primary EMN-S Targets	Mechanistic Pathway	Experimental Model/Cohort	Recent Evidence	Molecular/Cellular Outcomes	Clinical or Translational Signal	Development Stage	EMN-S Leverage/Strategic role	References
Indole-boosting synbiotics (ILA/IPA)	Encoder (microbiota → aromatic Trp catabolites) → Executor (glial AhR)	Targeted synbiotics and Trp co-substrates elevate indole-3-propionic acid (IPA) and indole-3-lactic acid (ILA). These ligands engage AhR in microglia/astroglia, suppress NF-κB/NLRP3, reduce amyloidogenic stress, and support synaptic/cognitive rescue.	(i) *Clostridium sporogenes* + xylan synbiotic in 5xFAD; (ii) Trp + ILA supplementation; (iii) Bifidobacterium strains in mouse + human biomarker studies.	Synbiotic raises IPA and improves cognition in AD mice (Li et al., 2024, Food and Function, preclinical). ILA reduces soluble Aβ via AhR signaling (Kim et al., 2024, BBI). Human/mouse work shows systemic ILA increases with psychobiotic regimens (translational).	↓ Soluble Aβ; microglial shift to reparative states; astroglial antioxidant responses; synaptic integrity preserved.	Serum ILA/IPA increase in humans on targeted psychobiotics; plausible encoder-level repair with paired stool/serum monitoring.	Preclinical + human translational (biomarker)	Recalibrates encoder signal quality; dampens executor amplification via AhR-mediated neuroimmune modulation.	Li et al., 2024; [196]; Kim et al., 2024 [197];
Bile acid augmentation (e.g., TUDCA)	Encoder Integrator interface (FXR/TGR5; gut–brain immune crosstalk)	Restores primary BA profiles linked to neuroprotection; activates FXR/TGR5 on immune/glial compartments; modulates cytokine tone and reshapes gut taxa; and aligns upstream encoder outputs with integrator stability.	Prospective MS cohort with serum metabolomics + MRI/OCT; randomized TUDCA trial in progressive MS assessing safety + immune/microbiome shifts.	Primary BAs at baseline predict slower brain/retinal atrophy; TUDCA is safe with immune + microbiome changes (Ladakis et al., 2025, *Med*; plus protocol/preprint).	BA modules show slower CNS atrophy; immune cell signatures and microbial composition shift with TUDCA.	Prognostic BA panel and feasible augmentation strategy support encoder stabilization and integrator re-biasing.	Prospective human + RCT (Phase 2–like safety)	Stabilizes encoder outputs and resets integrator–executor set points; seasonality-aware designs encouraged.	Ladakis et al., 2025; [77]
CB_2_-biased modulation	Integrator (eCBome) → Executor (microglia)	Selective CB_2_ agonism biases microglia toward reparative, phagocytic phenotypes with minimal CB_1_-linked psychotropic liability; reduces pro-inflammatory cytokines; and modulates α-syn aggregation/clearance.	(i) Brain cell-type mapping shows CB_2_ predominance in microglia; (ii) α-syn rodent models with CB_2_ ligands; (iii) human tissue/proteomic confirmation.	CB_2_ activation attenuates neuroinflammation and dopaminergic injury in PD-relevant models. Single-cell/cell-sort mapping refines CB_2_ distribution.	↓ Microglial activation; ↑ homeostatic/reparative microglia; ↓ α-syn pathology.	Rationale for executor-sparing anti-inflammatories without CNS side effects: combinable with encoder BA/SCFA repair.	Preclinical + human tissue mapping	Tunes integrator gain and mitigates executor overdrive; strong synergy with microbiome-directed strategies.	Grabon et al., 2024; [118]
PEA/OEA (nutraceutical NAEs)	Integrator (PPARα-linked lipid tone; NAE buffering)	Oral OEA elevates N-acylethanolamide pools and activates PPARα, attenuating inflammation/oxidative stress and improving metabolic indices; good safety/tolerability; PEA often co-formulated clinically.	(i) Double-blind RCT in PCOS (n ≈ 60–100) tracking inflammatory/oxidative markers and glycemic control; (ii) 2025 meta-analysis of RCTs on cardiometabolic endpoints.	OEA improves glycemic indices and reduces inflammatory/oxidative markers (Taghizadeh-Shivyari et al., 2024).	↓ CRP, TNF-α, IL-6; improved HOMA-IR and lipid profile (population-dependent).	Low-AE nutraceutical strategy with cross-domain relevance to neuro-immune settings as an integrator buffer.	Human RCTs + meta-analysis	Increases integrator buffering capacity; natural adjunct in multimodal EMN-S programs.	Taghizadeh-Shivyari et al., 2024; [198]
Microglial reprogramming (TREM2 agonism)	Executor (microglia)	AL002 (TREM2 agonist mAb) engages central microglial repair programs (phagocytosis, metabolic support). Dose-dependent ↓ CSF sTREM2 and ↑ microglia-recruitment markers show target engagement; however, Phase 2 INVOKE-2 was negative on clinical and biomarker outcomes.	Phase 1 (healthy volunteers/early AD biomarker PD); Phase 2 INVOKE-2 (early AD).	Phase 1: central target engagement and tolerability (Long et al., 2024). Phase 2: did not meet endpoints.	On-target biomarker shifts without clinical efficacy in Phase 2.	Mechanism validated; efficacy may depend on stage/combination/earlier windows.	Phase 1 completed; Phase 2 negative	Shapes executor waveform; likely needs pairing with amyloid/tau or encoder-level repair.	Long et al., 2024; [199]
CSF1R pathway modulation (e.g., pexidartinib/PLX3397)	Executor (microglial population dynamics)	CSF1R inhibition transiently depletes microglia with subsequent repopulation/reprogramming. Effects on α-syn pathology and neurodegeneration are model-, sex-, and timing-dependent (benefit or harm possible).	α-syn mouse/rat models; PLX3397/PLX5622 regimens; supportive mechanistic work across neuro-oncology/ALS.	α-syn inclusion-responsive microglia resist depletion by CSF1R blockade; sex-dependent microglial response to PLX3397. PLX3397 alters pathology trajectory in synucleinopathy model.	Microglial depletion/remodeling with variable α-syn outcomes and motor effects highlights precision timing.	Mechanistic tool with cautious translation; requires biomarker-guided dosing (e.g., TSPO/TREM2 panels).	Preclinical; early oncology neuro-applications	Executor “gain” reset; can reduce noise amplification if precisely timed; not disease-agnostic.	Stoll et al., 2024; [200]
CRISPR-enhanced phage/precision microbiota editing	Encoder (targeted bacterial deletion)	CRISPR-armed bacteriophages selectively debulk pathobionts via lytic killing + targeted DNA degradation, converting the microbiota into a programmable encoder with pathogen control and downstream metabolite re-balancing.	ELIMINATE Phase 2, Part 1 (LBP-EC01) in acute uncomplicated *E. coli* UTI; randomized open-label PK/PD lead-in with antibiotic arm; Part 2 blinded ongoing.	Part 1: Rapid, durable bacterial load reduction and acceptable safety with LBP-EC01 + TMP-SMX vs. antibiotic alone. Platform generalizable to gut targets; Part 2 ongoing.	↓ Pathobiont load; validated PK/PD; favorable safety.	Proof-of-concept clinical efficacy for programmable microbiota control; strong template for gut-directed encoder engineering.	Early clinical (Phase 2 Part 1 complete)	Transforms encoder from static to programmable; pairs naturally with metabolite and immune readouts.	Kim et al., 2024; [201]

## Data Availability

The original contributions presented in this study are included in the article. Further inquiries can be directed to the corresponding authors.
